# The effect and optimal parameters of electroacupuncture on post-stroke dysphagia: a meta-analysis of randomized controlled trials

**DOI:** 10.3389/fneur.2025.1673716

**Published:** 2026-01-12

**Authors:** Mengqi Yue, Xu Chen, Xiaolin Yang, Zirong Wang, Haocheng Yu, Haiqiang Wang, Qin Luo, Zongyan Ma, Yanchun Jiang, Yong Qiu, Jing Shi

**Affiliations:** 1 Second Clinical Medical College, Yunnan University of Chinese Medicine, Kunming, China; 2 Department of Acupuncture and Moxibustion, The First Affiliated Hospital of Yunnan University of Chinese Medicine, Yunnan Provincial Hospital of Traditional Chinese Medicine, Kunming, China; 3 Yunnan University of Chinese Medicine, Kunming, China

**Keywords:** electroacupuncture, meta-analysis, optimal parameters, post-stroke dysphagia, randomized controlled trials

## Abstract

**Objective:**

This research aimed to assess the effectiveness of electroacupuncture in treating post-stroke dysphagia (PSD) and to investigate the optimal stimulation parameters.

**Methods:**

We conducted a comprehensive review of eight databases: PubMed, Web of Science, Cochrane Library, Embase, CBM, CNKI, Wan Fang, and VIP. Randomized controlled trials (RCTs) on electroacupuncture for the treatment of PSD published between the inception of these databases and March 19, 2025, were integrated. The treatment’s effectiveness was evaluated using several outcome indicators, including the Video Fluoroscopic Swallow Study (VFSS) and the Water Swallowing Test (WST), which collectively assess swallowing function. Two independent reviewers performed risk of bias (ROB 2) evaluations, frequency of use for electroacupuncture parameter combinations through the R language (version 4.5.1), and data analysis using Review Manager (RevMan) version 5.4 and Stata SE 18.

**Results:**

This analysis encompassed a total of 30 RCTs that involved 2,290 patients. The research demonstrated an overall efficiency of (RR = 1.29, 95% CI: 1.23–1.34, *p* < 0.0001; *I^2^* = 13%, fixed-effects model). The mean differences for the various scales were as follows: VFSS (MD = 1.67, 95% CI: 1.26–2.09, *p* < 0.01; *I^2^* = 57%, random effects model). WST (MD = −0.75, 95% CI: −0.93 to −0.57, *p* < 0.01; *I^2^* = 54%, random effects model). Aspiration pneumonia (RR = 0.41, 95% CI: 0.25 to 0.68, *p* = 0.0005; *I^2^* = 8%, fixed effect model). Subgroup analysis revealed significant disparities between the various waveforms (Dense-sparse wave (Ds-W): RR = 1.58, *p* = 0.003 < 0.01) and the distinctions among the top three combinations of electroacupuncture parameter usage frequency (≥ 30 min + Ds-W, ≥ 30 min + Continuous Wave (C-W), and ≥ 30 min + Intermittent Wave (I-W) groups) were statistically significant (≥ 30 min + Ds-W: RR = 1.55, *p* = 0.03 < 0.05). In addition, there were no statistically significant differences between the other electroacupuncture parameter subgroups, which included the stimulation frequency and single treatment time (*p* > 0.05).

**Conclusion:**

Electroacupuncture, in conjunction with dysphagia training, is more effective than a solitary treatment in patients with PSD. Furthermore, applying waveforms with Ds-W might enhance the effectiveness of electroacupuncture for PSD. However, the higher risk of bias (ROB) in the included trials indicates that the quality of evidence for the outcomes of these assessments may be jeopardized. Thus, further high-quality clinical trials are urgently required to evaluate the efficacy and effectiveness of electroacupuncture parameters (waveforms) in the treatment of PSD, ultimately increasing the total level of evidence (PROSPERO registration number: CRD420251014881, https://www.crd.york.ac.uk/prospero/).

## 1 Introduction

The worldwide prevalence of post-stroke dysphagia (PSD) is about 45.06%, indicating that a significant number of stroke patients encounter dysphagia (1). PSD is a disorder characterized by damage to the nerve conduction pathways innervating the tongue, pharynx, larynx, and other brain regions, resulting in swallowing difficulties. It is a prevalent consequence of stroke, defined by the impaired transport of food from the mouth to the stomach, potentially resulting in pneumonia, dehydration, electrolyte imbalances, and nutritional deficiencies (2, 3). Furthermore, these complications have a significant impact on the physical health and social functioning of patients, in addition to their psychological health (4), ultimately significantly increasing the probability of a poor prognosis and even mortality.

Currently, PSD can be treated through various clinical approaches, including medication, swallowing function training, and dietary management. While these therapies may enhance patients’ swallowing function to a certain degree, the drugs are associated with negative consequences, including gastrointestinal pain, headache, and sleeplessness, and may lead to issues such as dependency on prolonged usage. Modifications to food texture and fluid consistency might influence patients’ nutritional intake, while prolonged tube feeding may increase the risk of infection (5, 6). While beneficial, not all individuals with dysphagia are feasible candidates for swallowing function training, as excessive or unsuitable training techniques may result in fatigue or damage to the muscles around the larynx and esophagus (7, 8). Traditional Chinese medicine possesses distinctive characteristics in the management of PSD. For instance, Radix clematis might augment the excitability of smooth muscle in the digestive tract (pharynx or lower and middle esophagus) and enhance its rhythmic peristalsis to alleviate local spasms, thereby partially regulating the swallowing function (9). Curcumin in ginger has been shown to enhance the swallowing reflex, raise salivary substance P levels, and activate TRPV1 receptors (10, 11). Nevertheless, Chinese medicine treatment is based on individual holistic identification, which means that practitioners must possess a high level of clinical experience and judgment to accurately identify the evidence pattern and create a corresponding treatment plan. Additionally, the therapeutic effect may fluctuate due to variations in quality control, species type, and geographic differences (12).

The World Health Organization (WHO) recommends acupuncture as a supplemental treatment for stroke (13). And electroacupuncture is a treatment that blends traditional acupuncture procedures with current electrical stimulation therapy to produce neuromuscular activation effects by stimulating specific acupoints. In terms of electroacupuncture’s efficacy in the treatment of PSD, all previously published meta-analyses, whether comparing electroacupuncture combined with swallowing training versus swallowing training alone (14, 15) or analyses using electroacupuncture alone and electroacupuncture in combination with other interventions (16), have yielded positive results for the treatment of PSD with electroacupuncture. The mechanism is multidimensional, involving nerve regeneration, swallowing reflex modulation, coordination of swallowing muscle movement, and neurotransmitter secretion regulation, such as 5-hydroxytryptamine, glutamate, and the N-methyl-D-aspartate receptor (17–19). In particular, electroacupuncture can boost local field potentials in the primary motor cortex’s non-infarct regions, stimulating pyramidal neurons to increase the effectiveness of motor signal transmission, as suggested by Cui et al. (20). Simultaneously, it enhances motor conduction velocity in the hypoglossal nerve and promotes electromyographic activity in the hyoglossus and genioglossus muscles, thereby relieving swallowing muscle paralysis. However, none of the meta-analyses described above have looked into the parameters that should be modified to improve the efficacy of electroacupuncture in the treatment of PSD. Acupuncture, a unique therapeutic approach in Chinese medicine, is frequently deviated from by different doctors and fails to maintain a specific level of homogeneity (21), resulting in low repeatability of acupuncture. Electroacupuncture, as a product of modern society, skillfully combines needling with electric current. On the one hand, it provides a new amount of stimulation for needling other than manipulation operation; on the other hand, this amount of electric current stimulation is manipulatable with a specific numerical value, which undoubtedly establishes a new point of reflection for the unification of the quantitative-effective relationship of acupuncture treatment. Different electroacupuncture parameters, such as waveform, frequency, stimulation time, and others, are closely related to clinical efficacy. However, in actual clinical electroacupuncture operations, due to a lack of understanding of electroacupuncture parameters, parameter adjustment is customary and arbitrary (22). Thus, in order to evaluate the clinical effectiveness of electroacupuncture for the treatment of PSD and investigate the ideal parameters of electroacupuncture for the clinical treatment of PSD, we performed a systematic analysis by retrieving RCTs of electroacupuncture for the treatment of PSD.

## 2 Methods

### 2.1 Protocol and registration

This study was conducted as a meta-analysis by the Cochrane Handbook for Systematic Reviews of Interventions and has been registered with PROSPERO under registration number CRD4420251014881. Our research strictly adhered to the Preferred Reporting Items for Systematic Reviews and Meta-Analyses (PRISMA) reporting guidelines. The PRISMA checklist is provided in S1 Appendix.

### 2.2 Literature search

We conducted a comprehensive search over eight databases, which included four English databases-PubMed, Cochrane Library, Embase, and Web of Science (WoS), and four Chinese databases-China Biomedicine (CBM), China National Knowledge Infrastructure (CNKI), Chinese Science and Technology Journals (VIP), and WanFang Database. The search timeframe spans from each database’s establishment until March 19, 2025 (the actual search completion date). Country, language, and publishing status are all unrestricted. We developed our search approach using the MeSH subject terms “electroacupuncture,” “stroke,” and “dysphagia,” with an emphasis on RCTs. The approach was customized to each database’s particular features. S2 Appendix lists specific search phrases and tactics for each database. Furthermore, we manually reviewed the reference lists of all included papers to figure out presumably relevant RCTs.

### 2.3 Inclusion and exclusion criteria

Studies incorporated into this analysis were not restricted by age, gender, ethnicity, or race, and the inclusion criteria were as follows: (1) Participants in the study had to be stroke patients according to the diagnostic criteria; (2) electroacupuncture was used as the intervention; (3) the control group received standard treatment (rehabilitation or medication); (4) the study design had to be an RCT; and (5) the study had to have recorded the rate of efficacy, the adverse effects of inhalation pneumonia, VFSS, and at least one of the WST outcomes.

Exclusion criteria: (1) nonrandomized controlled trials; (2) animal studies; (3) doctoral dissertations, conference papers, and case reports; (4) original text or full-text data were not available; and (5) review articles were excluded.

### 2.4 Study selection and data extraction

Two researchers independently conducted the initial screening based on the inclusion and exclusion criteria by reading the titles and abstracts. Then, they read the full texts to select the literature further. In case of any disputes, a third researcher was involved in the screening to retain the articles that met the criteria. After all the researchers reached a consensus on all the included data, the basic information, such as the first author’s name, publication year, subject status, sample size, and outcome indicators, was extracted. To obtain any missing or ambiguous information, the original writers of articles with incomplete data were approached by email or telephone.

### 2.5 Risk of bias in individual studies

All of the papers included were RCTs, and the risk of bias in these trials was assessed utilizing the Cochrane Risk of Bias Tool 2.0 (RoB 2). (1) The randomization procedure, (2) departures from the intended intervention, (3) cases of missing result data, (4) outcome measuring techniques, and (5) criteria for the selection of reported outcomes are the five primary areas in which this extensive instrument assesses potential biases. Each domain is categorized according to the risk of bias as low, high, or some concern. Two researchers independently evaluated each included study’s risk of bias in order to uphold strict methodological criteria. To guarantee an objective assessment, a third researcher resolved any disagreements or inconsistencies found during the independent assessment.

### 2.6 Data analysis

This meta-analysis’s primary objectives were to investigate the effectiveness of electroacupuncture in treating PSD and to identify the optimal electroacupuncture therapy parameters. RevMan 5.4 and Stata SE 18 were used for the meta-analysis. A *p*-value of less than 0.05 was deemed statistically significant, while a *p*-value of less than 0.01 was considered highly significant. The amount of effect for continuous outcome indicators was determined by the mean difference (MD), while the effect value for dichotomous variables was determined by the relative risk (RR) and its 95% confidence interval (CI). The χ^2^ test and *I^2^* quantitative analysis were used to evaluate inter-study heterogeneity: if *p* > 0.1 and *I^2^* < 50%, there was no significant heterogeneity between the included studies, and a fixed-effects model was used for meta-analysis; if *p* < 0.1 and *I^2^* > 50%, there was significant heterogeneity between the studies, and a random-effects model was used for analysis. Subgroup analyses were also carried out, including stimulation waveform, stimulation frequency, and single stimulation time. The electroacupuncture parameters of each study were plotted as Upset plots in R language (version 4.5.1), which visualized the frequency of use of the three parameters or the combination of two parameters. Upset plots provide an intuitive representation of frequency patterns across various intersection combinations. This study utilizes this visualization technique to clearly demonstrate the usage frequency of different parameter combinations, enhancing the clarity of data interpretation. In the Upset plots, the frequency of use is represented by the top bar, and the combination of the related parameters is indicated by connecting the dots below the bar, which correspond to the parameter names on the left. To investigate whether the parameters the researchers employed more frequently were also more beneficial, the electroacupuncture parameters were subgrouped into the top three frequency combinations in the literature. Additionally, we conducted efficacy result sensitivity analyses, eliminating low-quality trials where *I^2^* > 50%. Egger’s bias test and funnel plots were used to evaluate publication bias.

## 3 Result

### 3.1 Study selection

**Figure 1:**
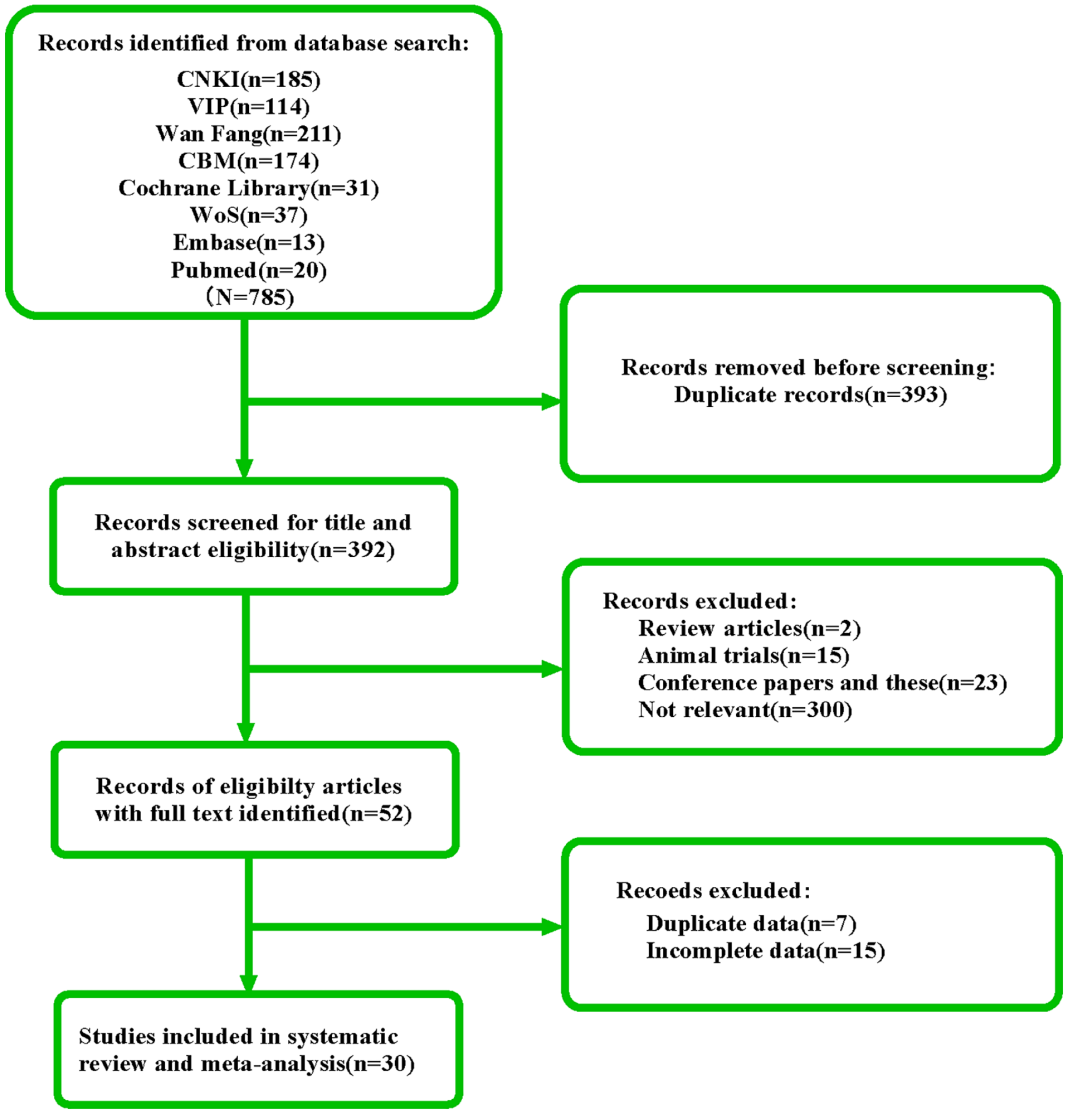
Literature screening process and results.

According to the search strategy, a total of 785 articles. The following table were retrieved. 393 duplicate publications, 23 conference papers and theses, 17 reviews, and animal experiments were discarded during the initial screening. By scanning through the titles, abstracts, and keywords of the literature, we discovered that 300 of them did not align with the study’s content. After reviewing the entire content, we discovered that 15 publications included redundant or insufficient information. Ultimately, this study contained 30 papers. The literature screening process is shown in Figure 1.

### 3.2 Study characteristics

There were 30 studies in total, involving 2,290 participants—1,146 in the treatment group and 1,144 in the control group—and all of them were RCTs. Twenty-eight of these articles were in Chinese, and two were in English. Every study was carried out in China. Regarding baseline variables, including age, length of illness, outcome indicators, and other fundamental data before enrollment, there were no statistically significant differences between the treatment and control groups. In the 30 investigations, the treatment group received electroacupuncture in addition to the treatment group, while the control group received traditional therapy and rehabilitation training.

Four evaluation instruments—VFSS, SWAL-QOL, WST, and SSA—were mostly employed by the 30 studies that comprised this study to gauge how well electroacupuncture worked to treat PSD. Eleven studies employed the G6805 model, the most common of the five electroacupuncture variants. The SDZ-II was used in four studies. The KWD-808I was used in four studies. Vitalstim was used in two trials. The HANS-100EI model was utilized in just one investigation. The electroacupuncture machine model utilized was not mentioned in the other eight trials. The waveforms were primarily Ds-W, C-W, and I-W; the stimulation frequency spanned from 0.5 HZ to 100 HZ; the treatment period ranged from 10 to 60 days, and the single treatment lasted 15 to 40 min. The detailed information and characteristics of the included studies are shown in Table 1.

**Table 1:** Basic information on the relevant literature is included in the study.

Study	Country	Disease course		Case		Age		Intervention		Main acupoints	EA parameter			EA-model
		TG	CG	TG	CG	TG	CG	TG	CG		Single time	Frequency	Wave	
Zhang et al. (36)	China	12.8 ± 4.6 days	11.6 ± 4.4 days	45	45	62.4 ± 9.6	61.2 ± 10.1	EA + ST + BT	ST + BT	ST9, CV23, EX-HN12, EX-HN13	30 min	30-80HZ	Symmetric bidirectional waves	Vitalstim
Wang et al. (58)	China	39.12 ± 20.79 days	37.50 ± 20.91 days	50	50	55.86 ± 10.30	53.66 ± 10.96	EA + ST + BT	ST + BT	CV23, GV16	15 min	2HZ	C-W	SDZ-II
Wang et al. (47)	China	NA	NA	40	40	67.4 ± 7.8	68.3 ± 9.3	EA + ST + BT	ST + BT	GB20, CV22, TTN	30 min	2HZ	C-W	HANS - 100E
Lan et al. (31)	China	14.47 ± 9.28 h	13.94 ± 8.67 h	38	38	56.86 ± 11.25	57.96 ± 11.76	EA + ST + BT	ST + BT	GB20, GB12, ST9, TE17, TTN	20 min	NA	I-W	KWD-808I
Ma et al. (27)	China	33.53 ± 20.86 days	31.91 ± 21.42 days	30	30	59.83 ± 11.31	59.01 ± 11.21	EA + ST + BT	ST + BT	TTN	40 min	NA	Ds-W	NA
Yang et al. (38)	China	8.34 ± 13.30 days	9.56 ± 14.91 days	35	35	67.91 ± 10.62	67.37 ± 9.75	EA + ST + BT	ST + BT	GB20, GB12, ST9, TE17, TTN	30 min	NA	Ds-W	G6805
Wang et al. (29)	China	25.03 ± 12.56 days	29.73 ± 12.01 days	30	30	62.47 ± 7.45	61.1 ± 7.74	EA + ST + BT	ST + BT	GB20, TTN	NA	2HZ	C-W	G6805
Wang et al. (45)	China	50.32 ± 30.88 days	46.26 ± 30.22 days	32	34	63.77 ± 9.32	68.14 ± 10.25	EA + ST + BT	ST + BT	GB20, GV16, TE17, TTN	30 min	NA	C-W	KWD-808I
Huang et al. (40)	China	42.6 ± 7.30 days	39.5 ± 7.30 days	32	30	60.50 ± 5.30	60.80 ± 6.20	EA + ST + BT	ST + BT	GB20, TTN	30 min	80-100HZ	Ds-W	G6805
Zhang et al. (37)	China	3.9 ± 0.5 months	3.5 ± 0.9 months	30	30	54.2 ± 4.3	53.7 ± 2.9	EA + ST + BT	ST + BT	TTN	30 min	50-100HZ	Ds-W	KWD-808I
Wang et al. (46)	China	NA	NA	30	30	NA	NA	EA + ST + BT	ST + BT	ST9	20 min	NA	C-W	G6805
Liu et al. (64)	China	3.11 ± 0.48 months	3.06 ± 0.51 months	47	47	59.28 ± 7.15	58.87 ± 7.21	EA + ST + BT	ST + BT	TE17, CV23	30 min	NA	C-W	SDZ-II
Yang et al. (43)	China	31.08 ± 5.60 days	30.12 ± 5.30 days	40	40	61.55 ± 8.45	60.78 ± 9.84	EA + ST + BT	ST + BT	GB20, TTN	30 min	2HZ	I-W	G6805
Li et al. (32)	China	26.89 ± 1.56 days	25.76 ± 1.53 days	50	50	42.6 ± 2.3	42.5 ± 2.2	EA + ST + BT	ST + BT	GB20, GV16	25 min	NA	Ds-W	SDZ-II
Jin et al. (34)	China	55 ± 3.4 days	55 ± 3.4 days	30	30	57.4 ± 7.6	58.3 ± 7.1	EA + ST + BT	ST + BT	ST9	30 min	2.5HZ	NA	NA
Zhao et al. (65)	China	NA	NA	50	50	NA	NA	EA + ST + BT	ST + BT	CV23, GV16	30 min	5HZ	I-W	G6805
Lu et al. (39)	China	17.85 ± 7.09 days	17.53 ± 5.62 days	15	15	60.56 ± 9.33	60.96 ± 8.25	EA + ST + BT	ST + BT	CV23	20 min	NA	Ds-W	Vitalstim
Zhang et al. (30)	China	NA	NA	44	44	55.6 ± 5.8	55.8 ± 5.2	EA + ST + BT	ST + BT	CV23, GB12, TE17	30 min	NA	Continuous sparse wave	G6805
Su et al. (48)	China	NA	NA	30	30	67.5	67.4	EA + ST + BT	ST + BT	GV26, GV16, GV23, GV20, GV15, GV14	30 min	NA	C-W	G6805
Cao et al. (52)	China	NA	NA	60	60	NA	NA	EA + ST + BT	ST + BT	GB20, EX-HN1	30 min	1.5HZ	I-W	NA
He et al. (42)	China	32 ± 15 days	27 ± 15 days	34	35	64 ± 6	69 ± 7	EA + ST + BT	ST + BT	EX-B1, GB20, CV23	30 min	5HZ	I-W	G6805
Chen et al. (33)	China	3.2 ± 1.4 months	3.5 ± 1.6 months	30	30	64 ± 5	65 ± 5	EA + ST + BT	ST + BT	GB20, GV16, TTN	30 min	NA	Ds-W	NA
Deng et al. (35)	China	NA	NA	69	69	58.2 ± 3.5	59.2 ± 3.9	EA + ST + BT	ST + BT	GB20	30 min	3-5HZ	C-W	NA
Zhang et al. (24)	China	39.48 ± 7.92 days	40.19 ± 8.35 days	59	57	58.47 ± 9.26	57.61 ± 9.83	EA + ST + BT	ST + BT	GB20, EX-HN12, EX-HN13	30 min	2HZ	C-W	NA
Fu et al. (25)	China	88.50(54.00,131.75) days	77.00d(37.50,100.00) days	24	24	65.67 ± 10.96	65.79 ± 8.05	EA + ST + BT	ST + BT	GB20	30 min	10HZ	C-W	NA
Tong et al. (40)	China	35 days	36 days	30	30	63	62	EA + ST + BT	ST + BT	CV23, EX-HN12, EX-HN13, GB20	30 min	NA	Ds-W	SDZ-II
Zang et al. (41)	China	81.42 ± 8.16 days	80.52 ± 9.04 days	50	50	65.27 ± 8.83	63.08 ± 7.65	EA + ST + BT	ST + BT	GB20, TTN	30 min	2HZ	I-W	NA
Li et al. (50)	China	44.57 ± 31.29 days	38.27 ± 32.92 days	30	30	63.93 ± 10.72	62.88 ± 10.15	EA + ST + BT	ST + BT	GB20, GV16, TE17, TTN	30 min	NA	C-W	KWD-808I
Jia et al. (51)	China	NA	NA	23	22	NA	NA	EA + ST + BT	ST + BT	CV23, GV20	30 min	NA	Ds-W	G6805
Meng et al. (44)	China	NA	NA	39	39	68.35 ± 4.74	65.52 ± 3.37	EA + ST + BT	ST + BT	GV26, GV16, GV23, GV20, GV15, GV14	30 min	NA	C-W	G6805

**Figure 2:**
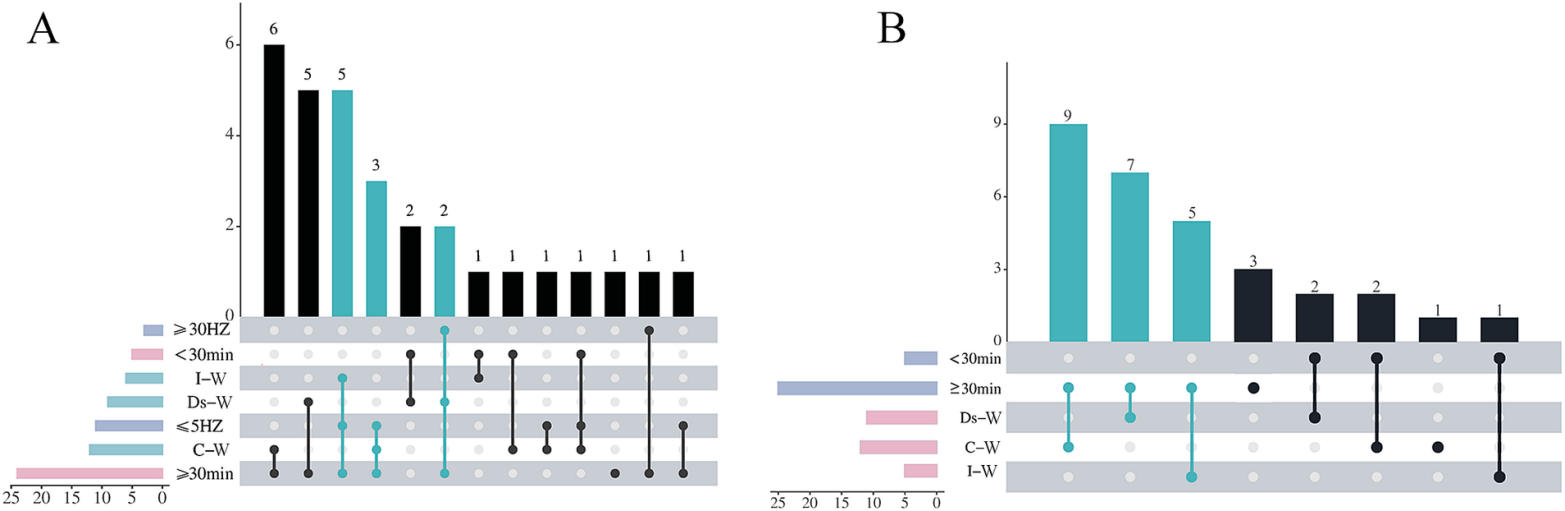
Upset plots on the frequency of combination use of electroacupuncture parameters. **(A)** Time + frequency + waveform combination usage frequency. **(B)** Time + waveform combination usage frequency.

We used the R language to create upset plots of the frequency of occurrence of the three parameter combinations of electroacupuncture (time + frequency + waveform) (Figure 2A), and then subgrouped the top three combinations (≥ 30 min + ≤ 5HZ + I-W, ≥ 30 min + ≤ 5HZ + C-W, and ≥ 30 min + ≥ 30HZ + Ds-W) to investigate whether the researchers used the higher frequency of the parameters with better efficacy. After conducting the subgroup analysis, we discovered that the difference in effect sizes between them was not statistically significant. Therefore, we had to conduct subgroup analysis using two parameter combinations (time + waveform, time + frequency, waveform + frequency) (Figure 2B). Finally, we observed that the difference in effect sizes was statistically significant for only one parameter combination (time + waveform).

### 3.3 Study design and risk of bias

**Figure 3:**
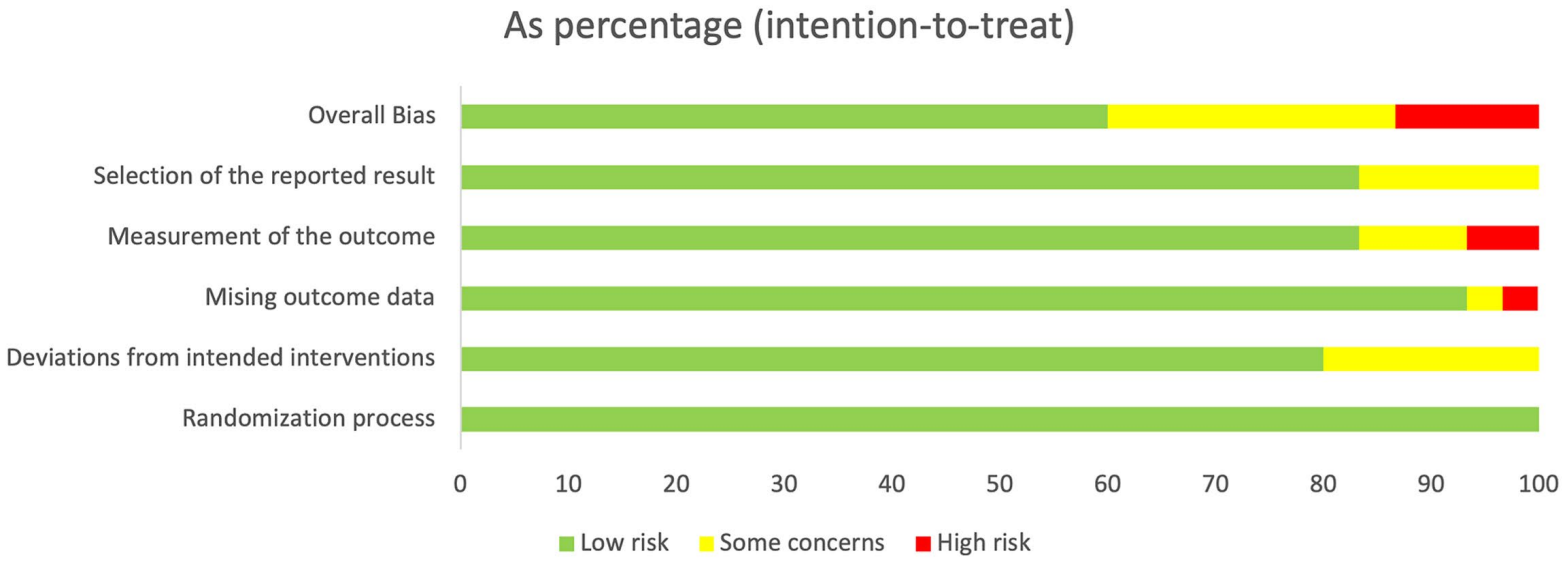
Assessment of risk of bias summary of included studies using the Cochrane tool.

**Figure 4:**
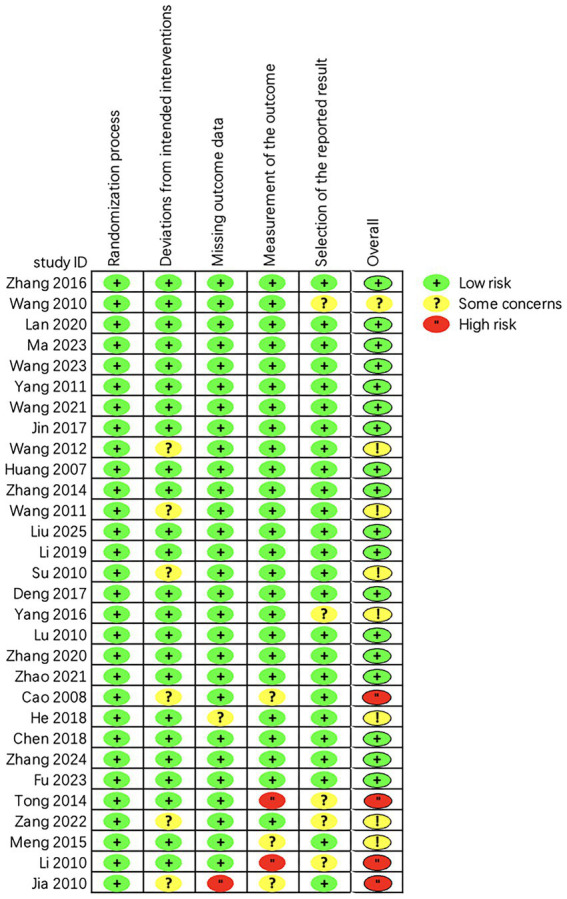
Assessment of risk of bias graph of included studies using the Cochrane tool.

The quality of the 30 randomized controlled trials included was rated as “low to moderate.” Due to the unique nature of the acupuncture treatment maneuver, all investigations were unable to blind either the operator or the participant. A total of 18 studies were classified as “low risk” (23–40). However, 8 studies were assessed as having a risk of “some concern” (41–48), and 4 studies were assessed as “high risk” (49–52). Regarding the examination of the randomization procedure, all of the research referenced randomized groups, and we noticed that the majority of the trials were randomized using the random number table approach. Figures 3, 4 show the detailed assessment.

### 3.4 Meta-analysis

#### 3.4.1 The total effective rate

**Figure 5:**
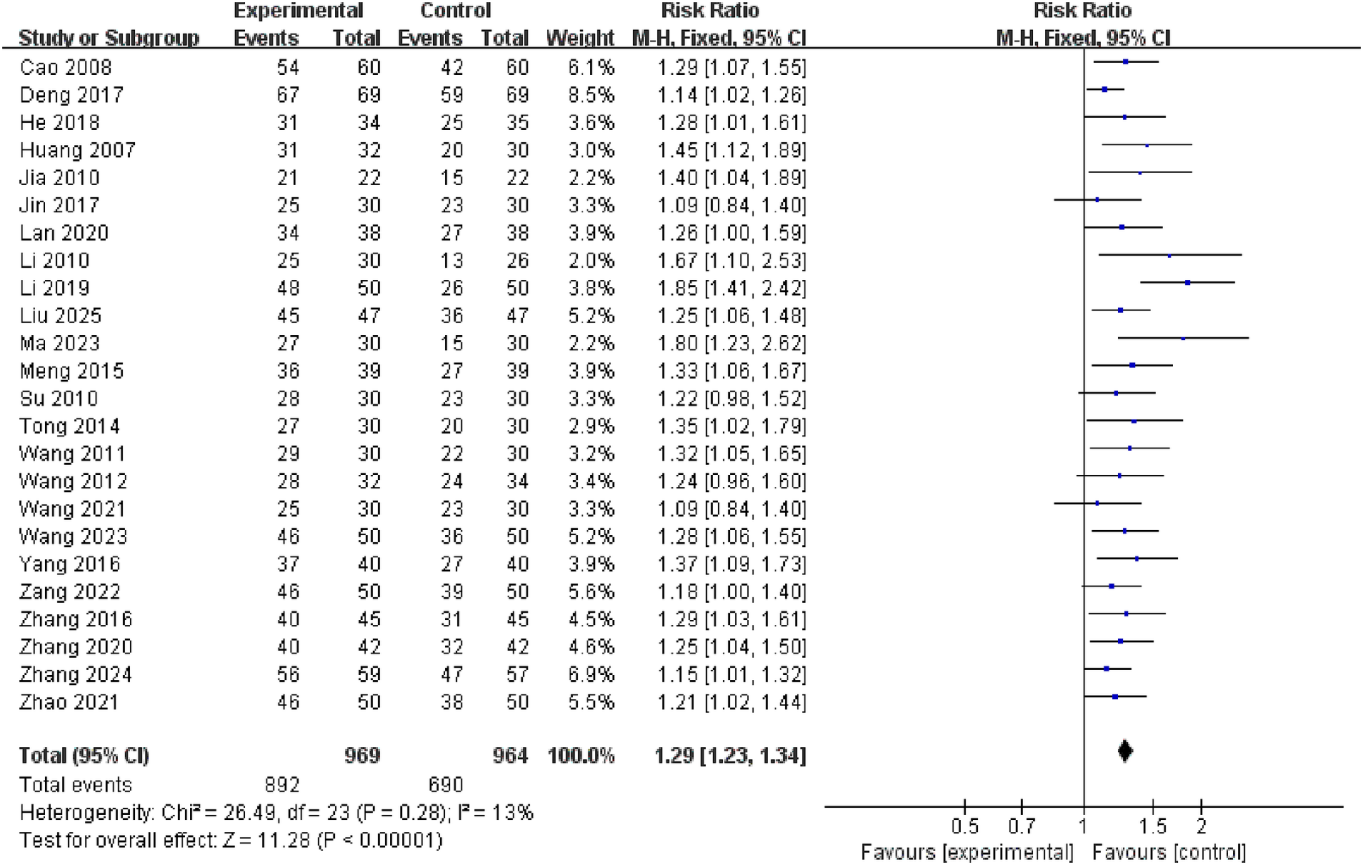
Forest plots of the total effective rate.

This research included 30 independent studies, 24 of which reported this specific result, and a subsequent meta-analysis using a fixed-effects model revealed that the efficacy of the electroacupuncture treatment group was significantly better than that of the control group (RR = 1.29, 95% CI: 1.23–1.34, *I^2^* = 13%, 24 studies, 1933 participants). Furthermore, the results of the heterogeneity analysis revealed that these findings were reliable. See the Figure 5 for details.

#### 3.4.2 Adverse aspiration to pneumonia reactions

**Figure 6:**
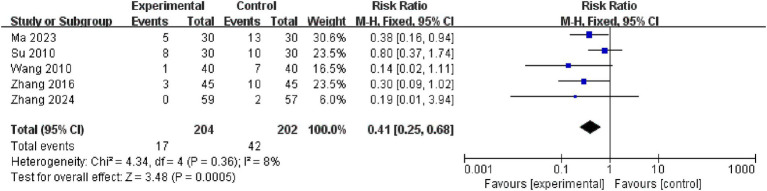
Forest plots of the adverse aspiration to pneumonia reactions.

This study included 5 studies on adverse reactions to aspiration pneumonia. The meta-analysis of the included studies was conducted using a fixed-effects model, which revealed that the electroacupuncture treatment group outperformed the control group in reducing the occurrence of adverse reactions to aspiration pneumonia, with a statistically significant difference (RR = 0.41, 95% CI: 0.25 to 0.68, *I^2^* = 8, 5 studies, 406 participants). Furthermore, the results of the heterogeneity analysis showed that these findings were reliable. See the Figure 6 for details.

#### 3.4.3 VFSS scores

**Figure 7:**
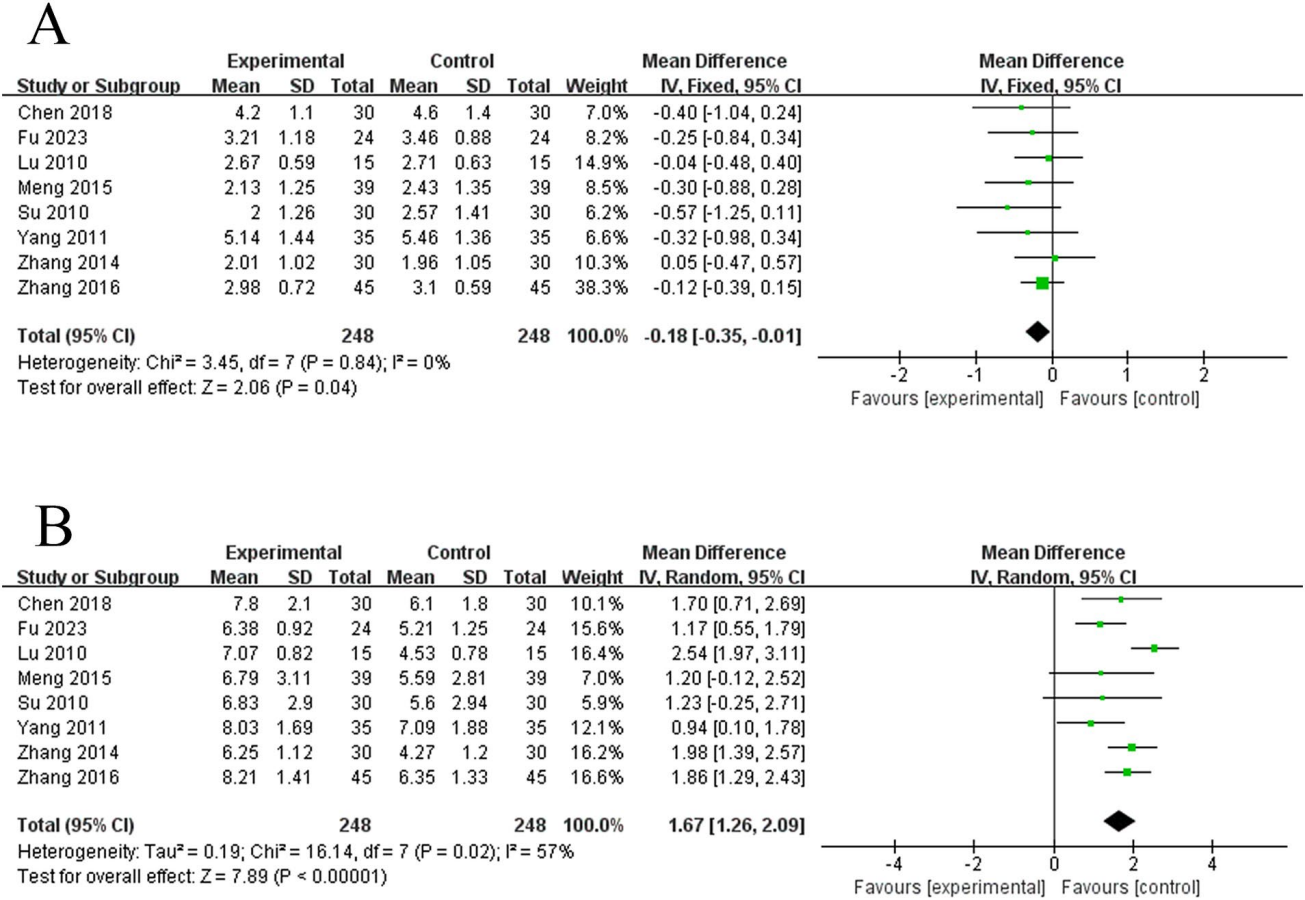
Forest plots of the VFSS scores. **(A)** Forest plot of VFSS scores for two groups before treatment. **(B)** Forest plot of VFSS scores for two groups after treatment.

This study encompassed 8 studies on VFSS scores. The meta-analysis of the baseline-period differences (effect sizes) in VFSS scores between their two groups using a fixed-effects model revealed that there was no difference in the two groups’ baseline VFSS scores (MD = −0.18, 95% CI: −0.35 to −0.01, *I^2^* = 0%, 8 studies, 496 subjects) (Figure 7A). The findings of the heterogeneity analysis revealed that these findings were reliable. And subsequent meta-analysis of post-treatment differences (effect sizes) in VFSS scores between the two groups using a random-effects model revealed that electroacupuncture was more effective than the control group in improving VFSS scores in patients with PSD (MD = 1.67, 95% CI: 1.26 to 2.09, *I^2^* = 57%, 8 studies, 496 subjects). Nevertheless, there was moderate heterogeneity among these studies (Figure 7B).

#### 3.4.4 WST scores

**Figure 8:**
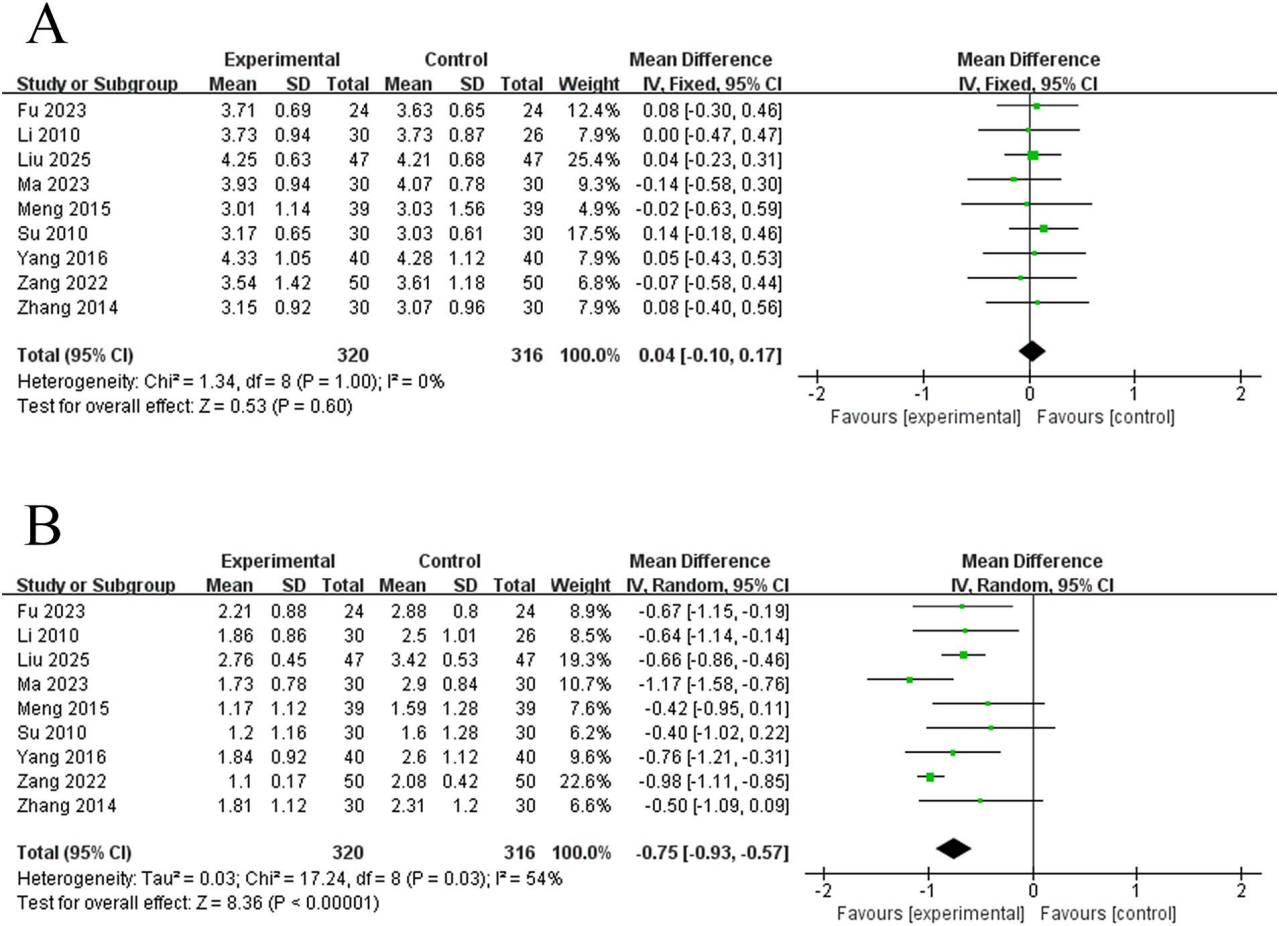
Forest plots of the WST scores. **(A)** Forest plot of WST scores for two groups before treatment. **(B)** forest plot of WST scores for two groups after treatment.

This study involved 9 studies on WST scores. The meta-analysis of the baseline period differences (effect sizes) of their WST scores between the two groups using a fixed-effects model revealed that there was no difference between the two groups’ WST scores at baseline (MD = 0.04, 95% CI: −0.10 to −0.17, *I^2^* = 0%, 9 studies, 636 subjects), and the results were robust, according to the heterogeneity analysis (Figure 8A). An afterward meta-analysis of post-treatment differences (effect sizes) in WST scores between the two groups using a random-effects model revealed that electroacupuncture was more effective than the control group in lowering WST scores in PSD patients (MD = −0.75, 95% CI: −0.93 to −0.57, *I^2^* = 54%, 9 studies, 636 subjects) (Figure 8B). However, there was moderate heterogeneity among these studies.

### 3.5 Analysis of subgroups

To investigate the ideal electroacupuncture parameters for PSD, we conducted a number of subgroup analyses. These included C-W vs. I-W vs. Ds-W waveforms, stimulation frequency (≤ 2.5 vs. > 2.5 HZ), single treatment time (< 30 vs. ≥ 30 min), and upset graphs utilizing the 2-parameter combinations of the top 3 frequencies (≥ 30 min + C-W vs. ≥ 30 min + Ds-W vs. ≥ 30 min + I-W).

#### 3.5.1 Single treatment time

**Figure 9:**
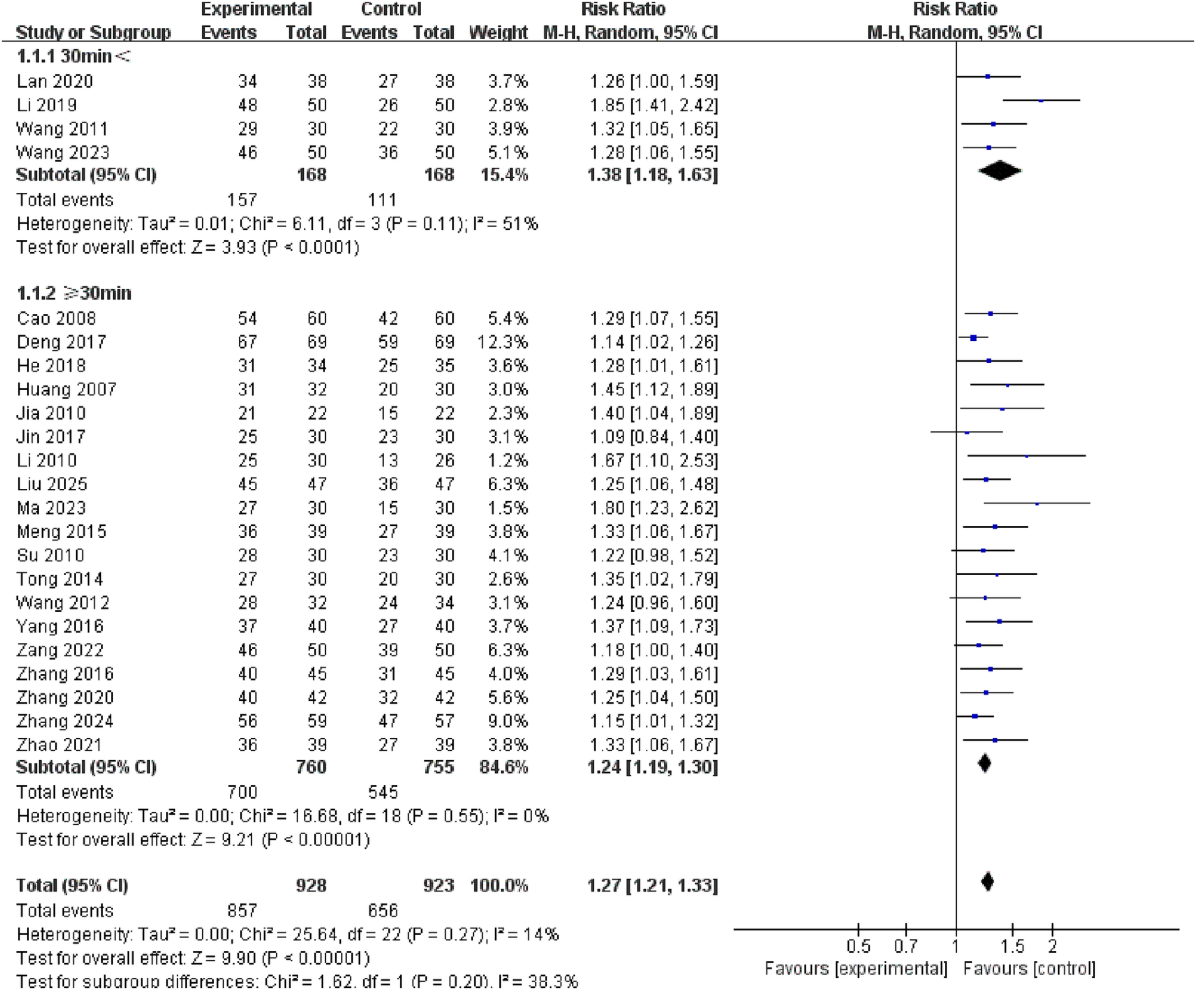
Forest plots of subgroup analysis of single treatment time.

A subgroup analysis of single treatment time revealed no significant difference between the < 30 min and ≥ 30 min groups (*p* = 0.20). Nonetheless, studies in the < 30 min group (RR = 1.38, 95% CI: 1.18–1.63, *p* < 0.00001) and the ≥ 30 min group (RR = 1.24, 95% CI: 1.19–1.30, *p* < 0.00001) showed higher effect sizes than the control group. See the Figure 9 for details.

#### 3.5.2 Waveform analysis

**Figure 10:**
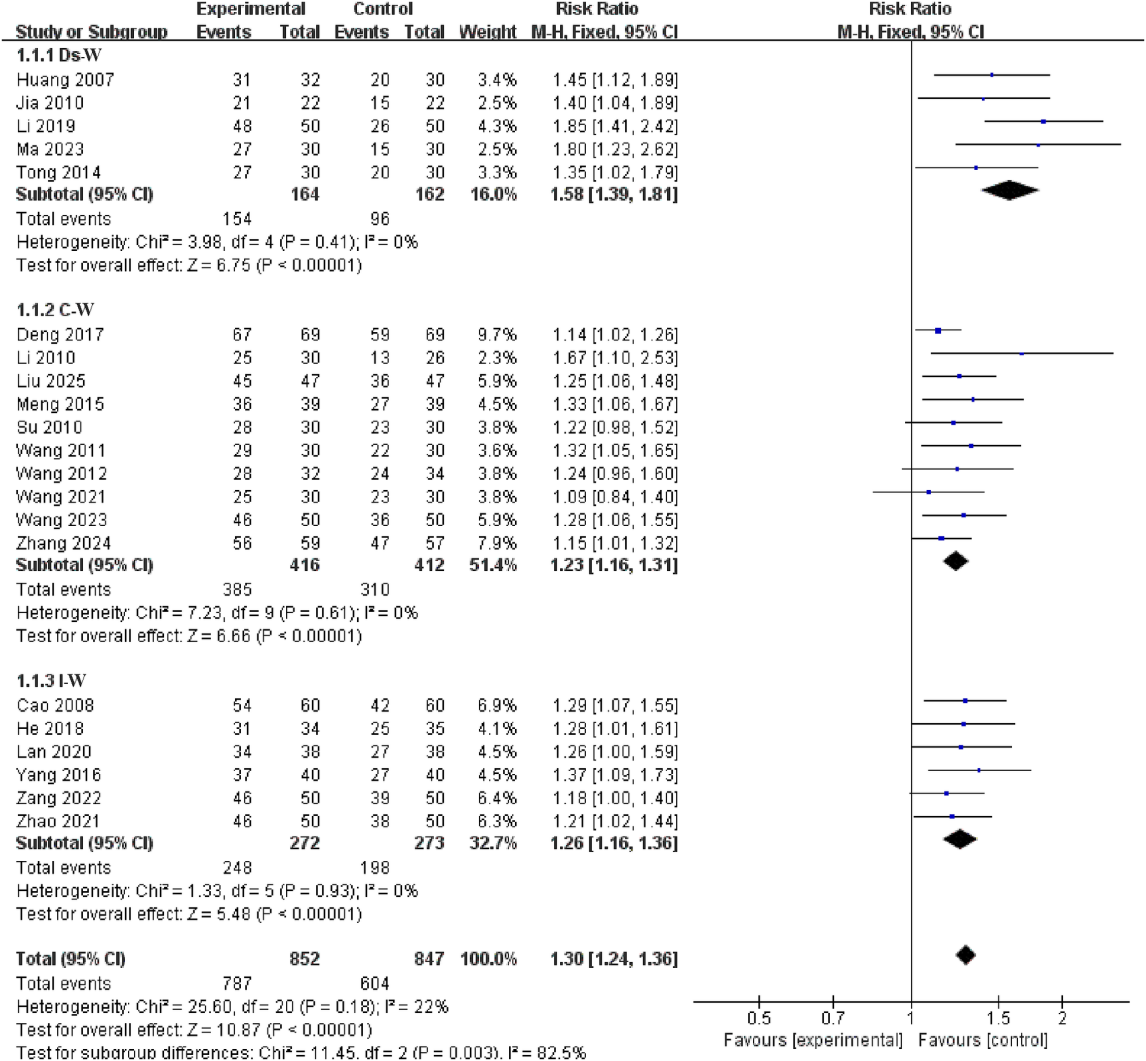
Forest plots of subgroup analysis of waveform.

Analysis of the different waveform subgroups showed significant variability among the C-W, I-W, and Ds-W groups (*p* = 0.003), with the highest study effect size in the Ds-W group (RR = 1.58, 95% CI: 1.39–1.81, *I^2^* = 0, 5 studies, 326 subjects), followed by the I-W group (RR = 1.26, 95% CI: 1.16–1.36, *I^2^* = 0, 6 studies, 545 subjects), and the C-W group ranked last in terms of study effect size (RR = 1.23, 95% CI: 1.16–1.31, *I^2^* = 0, 10 studies, 828 subjects). See the Figure 10 for details.

#### 3.5.3 Stimulation frequency

**Figure 11:**
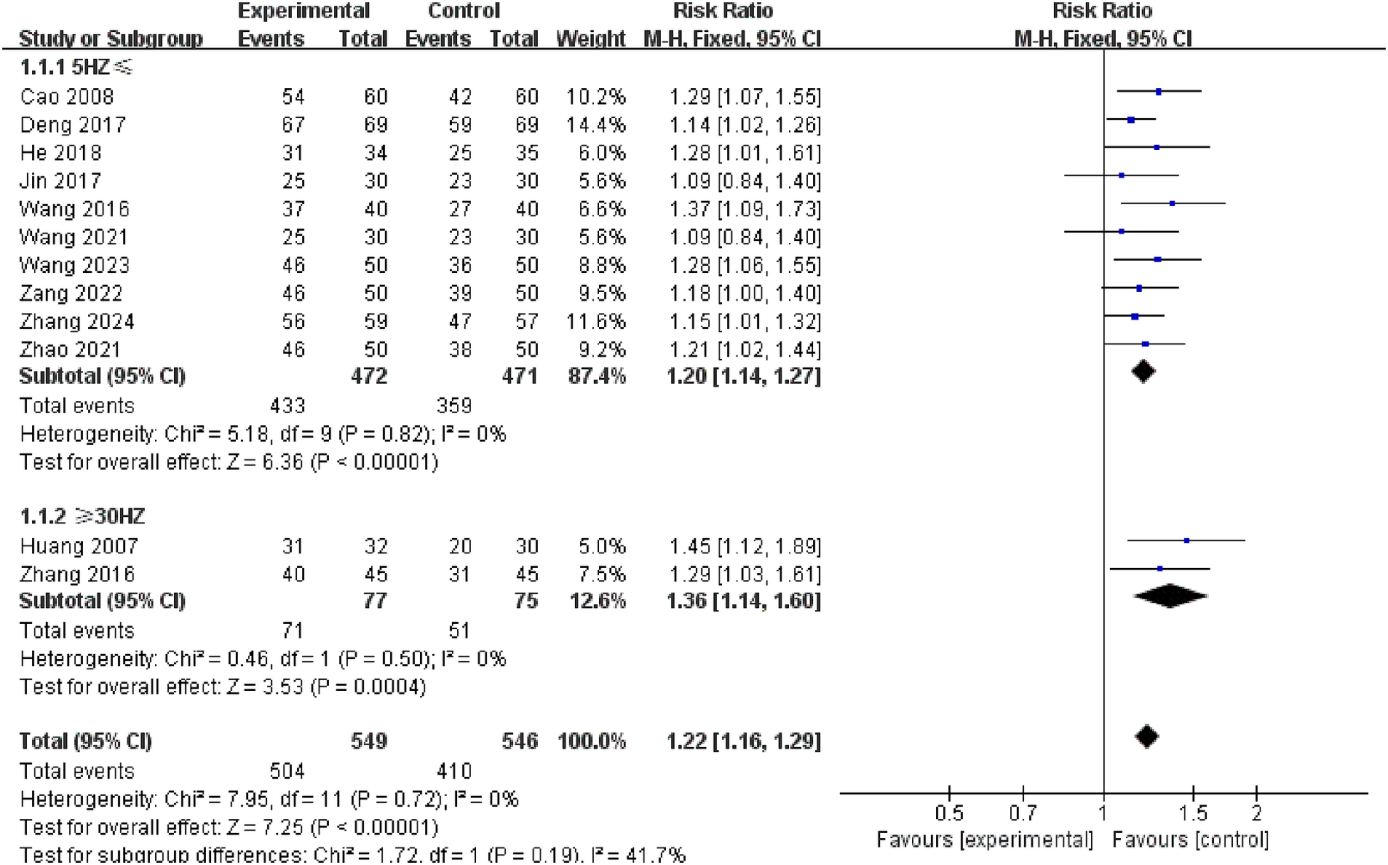
Forest plots of subgroup analysis of stimulation frequency.

Subgroup analysis of stimulation frequency indicated no significant difference between the 5HZ ≤ and ≥ 30HZ groups (*p* = 0.19). Studies in both the ≥ 30HZ group (RR = 1.36, 95% CI: 1.14 to 1.60, *p* = 0.0004) and the 5HZ ≤ group (RR = 1.20, 95% CI: 1.14 to 1.27, *p* < 0.00001) showed higher effect sizes than the control group. See the Figure 11 for details.

#### 3.5.4 Electroacupuncture parameter combination

**Figure 12:**
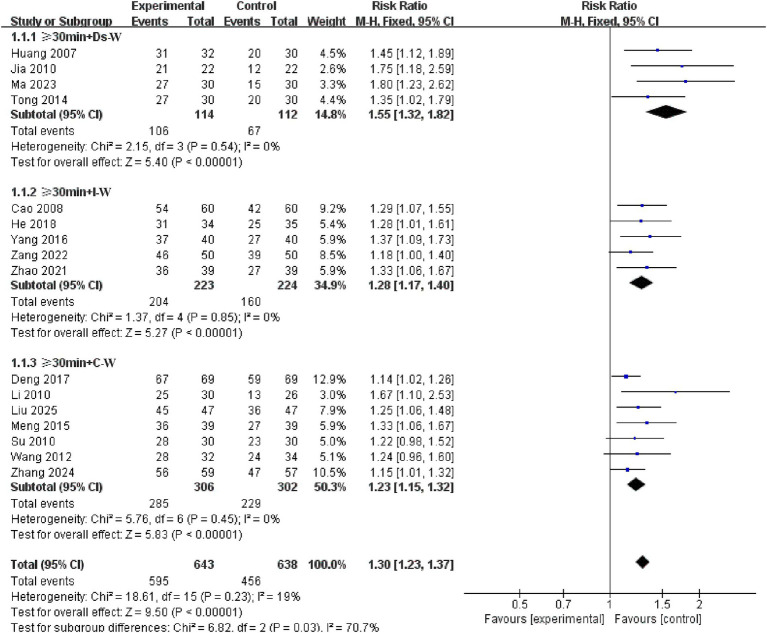
Forest plots of subgroup analysis of electroacupuncture parameter combination.

A subgroup study of the top 3 combinations of electroacupuncture parameter utilization revealed significant differences across time + waveform combinations (*p* = 0.03 < 0.05). The ≥ 30 min + Ds-W group had the highest study effect size (RR = 1.55, 95% CI: 1.32–1.82, *p* < 0.00001), followed by the ≥ 30 min + I-W group (RR = 1.28, 95% CI: 1.17–1.40, *p* < 0.00001) and the ≥ 30 min + C-W group (RR = 1.23, 95% CI: 1.15 to 1.32, *p* < 0.00001). See the Figure 12 for details.

### 3.6 Bias testing

#### 3.6.1 Effective rate

**Figure 13:**
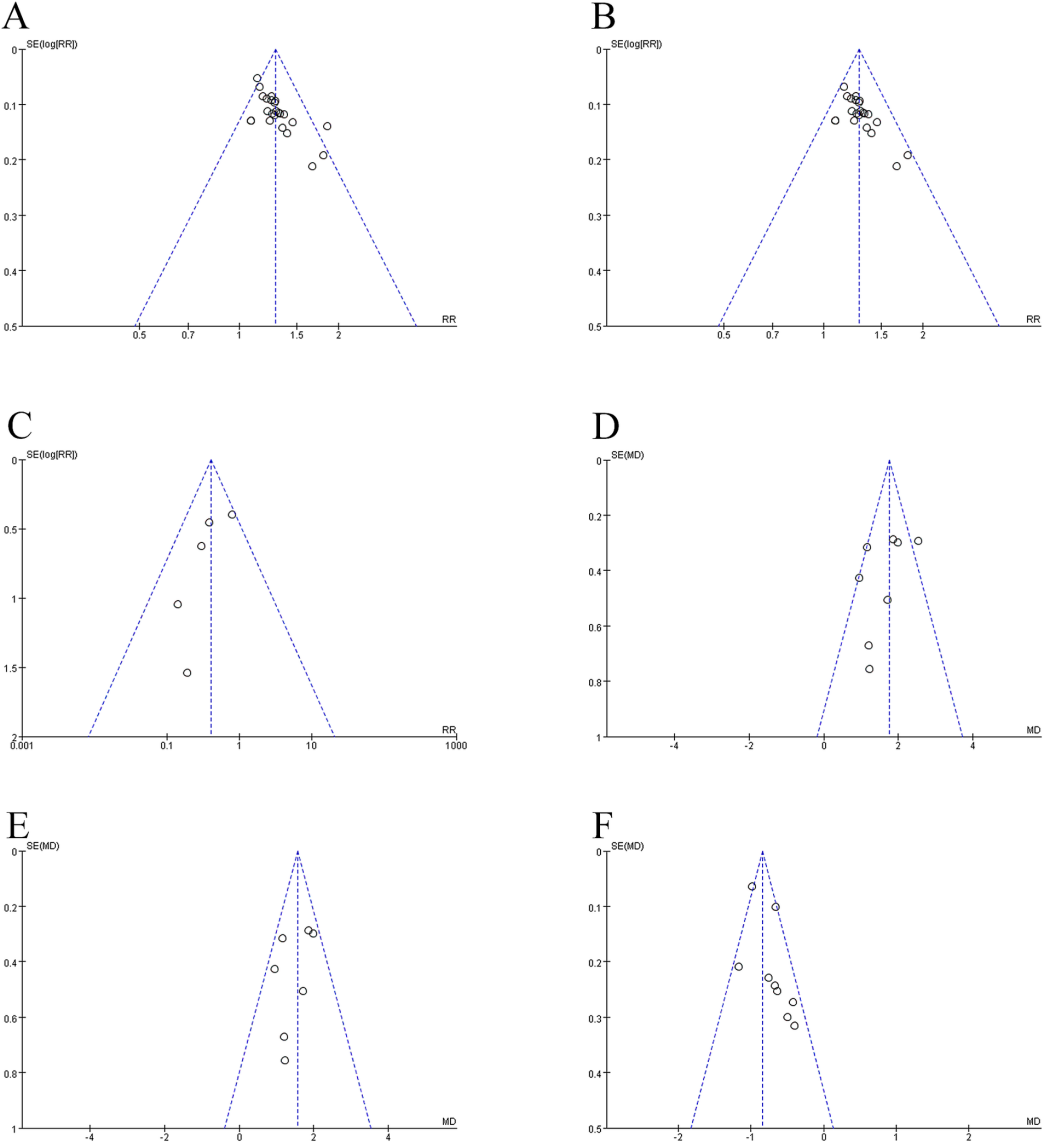
Summary of funnel plot. **(A)** Funnel plot of the effective rate. **(B)** Funnel plot of the effective rate after removing two studies. **(C)** Funnel plot of the adverse reactions to aspiration pneumonia. **(D)** Funnel plot of the VFSS scores. **(E)** Funnel plot of the VFSS scores after removing one study. **(F)** Funnel plot of the WST scores.

A test of bias on the 24 studies that recorded validity showed that the funnel plot was asymmetric, with 2 trials exceeding the 95% CI (Figure 13A). After excluding these 2 trials (Figure 13B), we observed *I^2^* = 0 < 50%, indicating that the reliability of electroacupuncture to improve PSD has been verified (RR = 1.28, 95% CI: 1.22–1.34, *I^2^* = 0, 22 studies, 1,695 subjects). Nonetheless, the funnel plot exhibited modest asymmetry. The clipping approach was used to rectify the asymmetric funnel plots, and after 5 rounds, the computer ultimately simulated the findings of 6 literatures, for a total of 28 literatures after clipping and without publishing bias (S5 Appendix). After clipping, the effect size of the combined 28 studies fell marginally (RR = 1.23, 95% CI: 1.17–1.28). No reversal of the results was seen. The conclusions of the existing meta-analysis may thus be deemed reasonably stable, and if additional findings or reports emerge in the future, they will not dramatically alter the meta-analysis’s results.

#### 3.6.2 Adverse reactions to aspiration pneumonia

The funnel plot analysis of 5 studies on adverse reactions to aspiration pneumonia revealed symmetry, with no studies being outside the 95% CI and *I^2^* = 8% < 50% (Figure 13C).

#### 3.6.3 VFSS scores

A bias test of the 8 studies with verified VFSS scores revealed that the funnel plot was asymmetric, with one study falling outside of the 95% confidence interval (Figure 13D). After removing one study (MD = 1.56, 95% CI: 1.28–1.85, *I^2^* = 17%, 7 studies, 466 individuals), we observed that *I^2^* = 17% was less than 50%, and the funnel plot revealed mild asymmetry (Figure 13E).

#### 3.6.4 WST scores

The funnel plot identified modest asymmetry in the analysis of 9 studies with recorded WST scores, with no research falling outside the 95% CI but an *I^2^* of 54% (Figure 13F).

Begg’s and Egger’s tests pinpoint the symmetry of the funnel plot, which indicates the presence of publication bias. Table 2 shows the findings of Begg’s and Egger’s bias tests. The Egger bias test for the incidence of adverse pneumonia (*p* = 0.128), VFSS (*p* = 0.323), and WST (*p* = 0.080) showed no publication bias. The Egger’s bias test for efficiency (*p* = 0.001 < 0.05) indicated publishing bias, but clipping and patching six papers revealed no publication bias (*p* = 0.796 > 0.05).

**Table 2:** Beeg test and Egger test for publication bias of outcome indicators reported in this study.

Outcome	Analysis stage	Begg (*p*-value)		Eegg (*p*-value)	
Effective rate	Before exclusion	0.000	<0.05	0.000	<0.05
	After exclusion	0.003	<0.05	0.001	<0.05
	Trimed	0.621	>0.05	0.796	>0.05
Adverse reactions of aspiration pneumonia	Before exclusion	0.142	>0.05	0.128	>0.05
VFSS	Before exclusion	0.322	>0.05	0.228	>0.05
	After exclusion	0.453	>0.05	0.323	>0.05
WST	Before exclusion	0.061	>0.05	0.080	>0.05

## 4 Discussion

The study includes 30 randomized controlled trials with 2,290 participants to assess the efficiency of electroacupuncture combined with swallowing training in treating PSD, measured by the effective rate, adverse effects of aspiration pneumonia, VFSS score, and WST score. Subsequent subgroup analyses were conducted based on various electroacupuncture parameters to identify the optimal conditions for treating PSD. The comprehensive efficiency assessment indicated that electroacupuncture in conjunction with swallowing training was superior to the alone swallowing training intervention (RR = 1.29, *p* < 0.01). Subgroup analysis revealed highly significant differences among the various waveforms (Ds-W: RR = 1.58, *p* = 0.003 < 0.01), and the distinctions among the top three combinations of electroacupuncture 2-parameter use frequency (≥ 30 min + Ds-W, ≥ 30 min + C-W, and ≥ 30 min + I-W groups) were statistically significant (≥ 30 min + Ds-W: RR = 1.55, *p* = 0.03 < 0.05). Moreover, no significant differences were observed among the other subgroups of electroacupuncture stimulation parameters (including treatment duration and stimulation frequency) (*p* > 0.05).

The significant efficacy of electroacupuncture in the treatment of PSD has been repeatedly validated by prior research (13, 14, 53, 54). In comparison to manual acupuncture, electroacupuncture offers advantages such as consistent stimulation intensity, excellent reproducibility, and independence from the clinician’s manual manipulation, thus being widely applied in clinical practice. Ye et al. (55) employed electroacupuncture intervention to assess neuronal discharge and c-Fos expression in SD rats, confirming that electroacupuncture can modulate swallowing function by activating swallowing-related interneurons in these rats. You et al. (18) demonstrated that electroacupuncture could promote reflexive swallowing activity by increasing the expression of 5-HT1A in the nucleus tractus solitarius by comparing the expression of 5-HT1A receptors in the nucleus tractus solitarius with that in the nucleus tractus solitarius after electroacupuncture intervention and the injection of 5-HT1A antagonist after electroacupuncture intervention. Jin et al. (56) discovered that electroacupuncture may enhance the sensory conduction of the glossopharyngeal, trigeminal, and vagus nerves, as well as activate the pharyngeal paralytic muscle groups by applying electroacupuncture to the supraglottic muscle groups of patients, thereby ameliorating PSD symptoms. The effectiveness of electroacupuncture for PSD is unequivocal, with its mechanism rooted in neuroanatomy and neurophysiology. Nonetheless, the majority of experimental research utilizing animal models in the literature predominantly involves small, healthy rats or mice. Moreover, there is a deficiency of research and innovation about PSD mouse modeling techniques. Furthermore, PSD is common among the elderly, and the majority of patients present with comorbidities; thus, further research should consider the variations in age and comorbidities within the model. The majority of studies concentrated on the immediate effects of electroacupuncture, both pre- and post-treatment, and did not investigate the temporal effects of electroacupuncture. Future research could address this gap to elucidate the mechanisms underlying the long-term effects of electroacupuncture. The stimulation volume of acupuncture in these experiments varied, which can be enhanced in future research. The degree of acupuncture stimulation is intimately linked to treatment efficacy (57, 58), a concept referred to as the acupuncture dose-effect connection (59), which incorporates stimulation intensity as a variable.

From the standpoint of electroacupuncture stimulation dose, two parameters initiate the stimulation dose: the stimulation location and the stimulation intensity. This study did not investigate this in groups, mostly due to the lack of variability and completeness of the data. Primarily, regarding stimulation points, the majority of the selected points in the studies we incorporated concentrated on the pharynx, with the Tongue Three-needle being the most emblematic. The upper Lianquan point (CV23) was selected as the first needle, located in the depression between the hyoid bone and the mandibular border. The second and third needles were positioned 0.8 inches to the left and right of this point (60), respectively. Anatomically, CV23 is innervated by branches of the upper transverse cervical nerve, the hypoglossal nerve, and the nerve to the mandibular glossopharyngeal muscle, among others. Acupuncture can modulate the activity of the autonomic nervous system and encourage the restoration of swallowing reflexes (60). It can also activate impaired nerve cells, expedite the repair of the swallowing reflex arc, and enhance the coordination of swallowing movements (61). The effectiveness of the Tongue three-needle treatment for PSD has been validated by expert consensus (62) and meta-analysis (63, 65). The literature reviewed in this study indicated that the majority of stimulated acupoints were located in proximity to the Tongue Three-needle, revealing no substantial differences in acupoint selection; therefore, we did not conduct a subgroup analysis. Conversely, it may be more significant to compare the Tongue Three-needle with the temporal three-needle. ① Prof. Jin Rui established both needles and are frequently utilized in conjunction (64); ② The temporal three-needle target acupuncture points are located 2 inches vertically from the ear tip to the hairline, and 1 inch horizontally in front of and behind the ear, categorizing them as head needles (65); ③ The temporal three needles, classified as head needles, have also been referenced in the guidelines (66). Investigating the efficacy and mechanisms of action of Tongue Three-needle and temporal three-needle as local and scalp stimulation, respectively, may enhance the effectiveness of PSD in the future. Regarding stimulation intensity, the majority of the research we incorporated was uninformative. We assert that two primary factors contribute to this situation: ① The requisite stimulation intensity in acupuncture is characterized by a localized aching and swelling feeling, referred to as “De qi” (67). Consequently, if a uniform stimulation strength is mandated, it is unfeasible for each patient to attain the state of qi, thereby inhibiting acupuncture from delivering its intended therapeutic impact. ② Individual pain sensitivity varies, and it is unequivocally immoral for the experimenter to overlook the patient’s pain experience to attain a uniform stimulation level. A study (68) investigated the best electroacupuncture treatment for knee osteoarthritis utilizing the artificial intelligence Apriori algorithm, revealing that the range of stimulation intensities required to attain therapeutic efficacy was broader in male patients than in female patients. This further substantiates that employing a consistent stimulation level for all patients would be illogical and irrational. This meta-analysis focuses on the waveform, frequency, and duration of electroacupuncture stimulation, excluding the intensity element.

The subgroup analysis of treatment duration revealed that both < 30 min and ≥ 30 min exhibited substantial effect sizes; however, no significant differences were observed between the two groups. This result is in line with earlier research. For example, Ye et al. (69) conducted low-frequency electroacupuncture treatments of 10, 20, and 30 min on patients with PSD. There were no statistically significant variations in the overall treatment effectiveness rate or the incidence of lung infection between the three groups. Other research, however, has given opposing viewpoints, proposing that decreasing needle retention time could improve treatment efficacy. For example, Luo et al. (70) found that the 15-min electroacupuncture group was more effective than the 30-min group in treating PSD patients. The disparities in results between studies can be attributable to major variances in their research methods, including variations in inclusion criteria for study individuals, treatment cycles, and electroacupuncture settings. This meta-analysis has a larger number of participants (*n* = 336 for the <30 min group and *n* = 1,515 for the ≥30 min group), which may increase the reliability of its results. The results of this study have substantial reference value from the standpoint of clinical practice: When applying electroacupuncture therapy to patients with PSD, practitioners can choose flexible needle retention times between 15 and 30 min based on real-world conditions (such as patient tolerance, patient flow during treatment periods, and treatment efficiency), without having to place an undue emphasis on long retention times. This flexible scheduling strategy improves treatment compliance by lessening patient discomfort brought on by extended bed rest or therapy. It is especially appropriate for elderly patients, people with weak constitutions, or people who cannot stand being in one position for long periods of time.

The subgroup analysis of various waveforms revealed that Ds-W, I-W, and C-W exhibited substantial effect sizes, which were markedly different across the three groups. Ds-W exhibits the most excellent effect size. The core parameters of electroacupuncture waveforms (frequency, pulse width, and current intensity) directly determine their regulatory effects on the neuromuscular system (71). Ds-W, a composite waveform that alternates between dense waves (high-frequency continuous pulses) and sparse waves (low-frequency intermittent pulses) (72), has the particular benefit of concurrently accomplishing both “repair effects” and “regulatory functions.” This dual effect establishes a special physiological mechanism for restoring neural function in dysphagic patients. Previous research has verified (73) that electroacupuncture with Ds-W intervention in mice with PSD resulted in considerable healing of injured neurons in the brainstem nucleus ambiguus. As an essential “execution center” for swallowing movements, functional restoration of the nucleus ambiguus regulates the contraction timing of major swallowing muscle groups such as the tongue, pharynx, and larynx (74), effectively improving core PSD symptoms such as post-swallowing food residue and aspiration. Further studies reveal (75) that electroacupuncture Ds-W therapy may encourage the release of several endogenous opioid peptides, such as *β*-endorphin and enkephalin, resulting in synergistic benefits. Numerous neuronal nuclei in the brainstem are involved in controlling the swallowing reflex. In order to optimize the signaling pathways between the swallowing center and peripheral muscle groups, these endogenous opioid peptides may act on receptors in brainstem neurons to modify neuronal excitability and neural signal transmission efficiency. Notably, the Ds-W holds a distinguishing feature over other electroacupuncture waveforms: it efficiently overcomes the “needle sensation tolerance phenomenon.” This problem refers to the temporary loss or absence of needle sensation shortly after patients receive electroacupuncture stimulation (76), and Ds-W was designed to solve this issue (77). Previous research has verified (78) that the anti-needle sensation tolerance effect of Ds-W is region-specific, with higher efficacy in the head and face than in other body regions. Since the neck and head area is the primary focus of electroacupuncture treatment for PSD, the alternating current characteristics of Ds-W applied to this area (such as pertinent neck muscle groups) can trigger rhythmic contractions in the target muscles. Ds-W improves the coordination of swallowing-related muscle groups because they are less likely to cause tolerance in the body than other waveforms.

In conclusion, on account of its numerous benefits in brainstem nucleus nerve repair, signal conduction regulation, and particular anti-tolerance effects in the neck and head area, along with markedly increased therapeutic efficacy, electroacupuncture Ds-W therapy exhibits wider clinical applicability and superior value in PSD treatment. I-W is a rhythmic wave pattern characterized by alternating intermittent and continuous waves, which enhances the excitability of muscle tissues and effectively stimulates contraction in transverse muscles. For instance, in the context of shoulder subluxation improvement, I-W is more effective than Ds-W and C-W (79). A continuous wave is a singular pulse created by amalgamating many methods, resulting in a unified effect that facilitates bodily adaptation with minimal impact. A study evaluating the alteration of needle feeling in electroacupuncture at the Quanliao point using Ds-W and C-W revealed that the rate of diminishing needle sensation in the C-W group was much more rapid than in the Ds-W group (77). This feature suggests that for certain patients who are sensitive to electroacupuncture stimulation and struggle to endure it, opting for C-W may be an appropriate alternative.

The subgroup analysis of stimulation frequency revealed that both the ≤ 5 Hz and ≥ 30 Hz groups had substantial effect sizes. However, the effect sizes did not differ significantly between the two groups. Based on the differences in brain activation regions between low and high frequencies, and combined with the stimulation frequency distribution characteristics derived from relevant studies, we categorized frequencies into a low-frequency group (≤ 5 Hz) and a high-frequency group (≥ 30 Hz). The aforementioned results show that there was no statistically significant difference in effect sizes between the high-frequency and low-frequency electroacupuncture groups. Significantly, current research indicates (80) that different electroacupuncture frequencies selectively activate different brain regions: 100 Hz high-frequency electroacupuncture primarily targets somatosensory areas, whereas 2 Hz low-frequency electroacupuncture primarily activates the ventral thalamus, basal thalamus, entorhinal cerebral cortex, and motor-related areas, with more pronounced activation effects on the brainstem (81). This finding was thoroughly corroborated in basic experiments: in a post-stroke dysphagia (PSD) rat model, 2 Hz electroacupuncture reduced neurological impairment and encouraged repair by upregulating c-fos neuronal expression in the brainstem reticular formation (73, 82); Simultaneously, it activates two neuronal pathways centered on the brainstem nucleus tractus solitarius (NTS)—the “M1-PBN-NTS” and “NTS-VPM-S1” pathways—to improve swallowing function (61, 83). In contrast, Yao et al. (84) study observed that 50 Hz and 100 Hz high-frequency electroacupuncture had much lower effects on primary motor cortex neurons than 2 Hz low-frequency electroacupuncture (as evaluated by c-Fos expression). The brainstem, known as the “central pattern generator” of the swallowing reflex (85), acts as the primary regulatory center for swallowing motions. The higher responsiveness of the brainstem to 2 Hz electroacupuncture provides distinct benefits for PSD treatment. Beyond central mechanisms, 2 Hz electroacupuncture’s efficacy in improving PSD may involve peripheral regulatory pathways, such as increasing peripheral blood perfusion by activating the TRPV1 signaling pathway (86, 87). Additionally, we discovered that three clinical studies comparing frequencies for electroacupuncture treatment of PSD (88–90), all of which verified that 2 Hz low-frequency electroacupuncture is more effective than 100 Hz high-frequency electroacupuncture. This result runs counter to our study’s conclusion. We hypothesize that this disparity could be caused by the fact that our analysis only included two high-frequency electroacupuncture studies, which would have resulted in insufficient statistical power. Based on the above analysis, it is advised that future studies include more high-quality head-to-head comparisons of high- and low-frequency electroacupuncture for PSD. If conditions allow, a special meta-analysis might be conducted on trials in which the treatment and control groups received high- and low-frequency electroacupuncture, respectively. This approach would provide higher-quality evidence-based support for improving clinical treatment strategies by raising the standard of evidence for comparing these frequency effects.

From the subgroup analysis of the top 3 combinations of frequency of use of electroacupuncture 2 parameters, the ≥ 30 min + Ds-W, ≥ 30 min + C-W, and ≥ 30 min + I-W groups showed significant effect sizes, and the difference in effect sizes was statistically significant among these three groups. The parameters of electroacupuncture are interdependent, influencing one another, and various combinations yield distinct electroacupuncture results (91). The primary objective of this subgroup analysis was to explore potential efficacy differences across various parameter combinations. Specifically, we first visualized the most frequently used parameter combinations in the included studies using Upset plots, and then further evaluated whether these commonly adopted parameters exhibited superior therapeutic efficacy. Nevertheless, owing to the inadequate documentation of the parameters utilized in the referenced literature, a limited number of studies reported all three parameters concurrently, and no statistically significant changes in effect sizes were observed in the subgroup comparisons of the three parameters. The identification of a statistically significant difference in effect sizes within the subgroup comparison of two parameters (time and waveform) is not regarded as an additional finding, as the analysis of the waveform subgroups has previously demonstrated a significant difference in effect sizes among various waveforms. A targeted orthogonal test would effectively examine the efficacy of various combinations of electroacupuncture parameters. Kuai et al. (92) utilized an orthogonal experimental design to examine the analgesic effects of various frequencies, waveforms, and current intensities on rats with adjuvant arthritis, ultimately identifying the best combination of analgesic parameters. A three-factor, two-level orthogonal test examining time, waveform, and frequency will be done to compare the efficacy of various electroacupuncture parameters for PSD. Concurrently, the researchers must document the operational methodologies, including the parameters, which can demonstrate the study’s legitimacy and promote the advancement of the discipline.

The proposed mechanism of electroacupuncture for PSD can be categorized into four fundamental aspects: ① Stimulation of pertinent nerve fibers, including the glossopharyngeal and vagus nerves, to enhance nerve fiber regeneration and increase the coordination and flexibility of pharyngeal muscles. ② By means of peripheral stimulation to enhance local blood circulation, facilitate the healing of the central nervous system, and expedite the reconstruction of the stroke unit. ③ Activate the impaired central cortical tissues to enhance neuronal discharge, hence increasing neurotransmitter release to stimulate pharyngeal-associated muscle groups. ④ Enhance the rebuilding and regenerative capabilities of the reflex arc, expedite the transmission of nerve impulses, and restore its neural function. Regarding the ideal parameters for electroacupuncture treatment of PSD, it should be pointed out objectively that this study has limitations. Consequently, it is not possible to directly draw the conclusion that “Ds-W are statistically significantly preferred waveforms” with definitely optimal parameters. The following are the precise causes: First, there is potential for improvement in the statistical test efficacy due to the small sample size of included literature, which may impact the stability and generalizability of statistical results. Second, optimizing electroacupuncture parameters essentially entails the synergistic adaptation of three fundamental elements: time, frequency, and waveform. Instead of independent selection based on a single dimension, optimal parameters should represent the best mix of these three aspects. Even though some of the study’s parameters did not satisfy the criteria for statistical significance, this does not mean that altering those parameters would have the same therapeutic effects. Because the effect sizes of the other parameter subgroups exhibited variations among the individual parameters. The absence of statistically significant effect sizes is, in our view, mostly attributable to the inadequate number of pertinent studies and the insufficient reporting of electroacupuncture parameters within those studies. It is advisable to consider the results of the subgroup analysis of other factors in this study alongside the clinical trials of various electroacupuncture parameters for the treatment of PSD. The duration of a single treatment may range from 15 to 25 min, with a stimulation frequency characterized by low-frequency electroacupuncture at 2 Hz. The primary stimulation sites may include the three needles on the tongue, in conjunction with pertinent acupoints in the pharynx or head, and the stimulation intensity should be calibrated to the patient’s maximum tolerance level. In the future, researchers may conduct comprehensive studies and investigations on the treatment of PSD utilizing electroacupuncture parameters, including orthogonal tests.

Several limitations to this study should be taken into account: First, because of the inherent properties of electroacupuncture treatment, blinding both the operator and the participants was impossible. This could lead to an overestimation of the actual effectiveness of electroacupuncture in individual trials due to subjective efficacy expectations among subjects and researchers. The overall impact size may lean toward “electroacupuncture being more effective” when similar results are analyzed in meta-analyses, jeopardizing the objectivity of conclusions. Second, there may have been selection bias in allocation because several of the included studies did not specifically define certain randomization techniques (such as centralized randomization or random number tables). This kind of bias could lead to positive bias in meta-analysis findings, which would undermine the validity of conclusions. Given the two types of bias indicated above, this study’s conclusions should be interpreted and applied with caution. Third, the majority of research did not fully document electroacupuncture parameters. Due to this shortcoming, subgroup analyses based on various parameter combinations are directly prohibited, restricting analyses to individual parameters alone. The reliability and reference value of subgroup analysis results are compromised by this method’s failure to take parameter interactions into account. Subsequent researchers should fully document the parameters when publishing the results of the trial, which not only reflects the completeness of the trial but also facilitates the development of the discipline. Fourth, the general acceptance of electroacupuncture needle therapy varies from individual to individual, and all the studies included in this analysis were conducted in China. This geographical limitation significantly diminished the generalizability of the meta-analysis results, which means that the study’s findings may be more applicable to patients in China and East Asia who have similar clinical situations. Finally, the subgroup analyses in this study chose to explore the effectiveness rate as the effect size, and the credibility of the results may not be as comparable as the comparison using the same swallowing scale score, which may be compared with the subsequent publication of a new relevant study.

## 5 Conclusion

Despite the suboptimal sample size and methodological quality of the 30 RCTs included in this study, we noted the outcome indicators before and after treatment, particularly the alterations in overall effectiveness, VFSS score, WST score, and adverse effects of aspiration pneumonia, indicating that electroacupuncture combined with swallowing training can improve PSD.

Meanwhile, this meta-analysis indicates that the selection of Ds-W may be more efficacious than C-W and I-W in the electroacupuncture therapy of PSD. The elevated risk of bias (ROB) in the included studies indicates that the quality of evidence concerning these evaluated outcomes may be undermined. Consequently, there is an urgent necessity for future high-quality clinical trials to evaluate the efficacy and effectiveness of electroacupuncture parameters (waveforms) in the treatment of PSD, thereby enhancing the overall level of evidence.
