# Supplementary material for: The effect and optimal parameters of electroacupuncture on post-stroke dysphagia: a meta-analysis of randomized controlled trials
Source: Front Neurol. 2026 Jan 12;16:1673716. doi: 10.3389/fneur.2025.1673716 (PMC12832666; doi:10.3389/fneur.2025.1673716)
Supplement: Supplementary file 1 [file Table_1.docx]

***Supplementary Material***

**Supplementary File 1 search strategy**

**Table 1:** PubMed Search

| **NO** | **Search Details** | **Results** |
| --- | --- | --- |
| #1 | "Stroke"[MeSH Terms] | 189,590 |
| #2 | "Deglutition Disorders"[MeSH Terms] | 62,746 |
| #3 | "Electroacupuncture"[MeSH Terms] | 5,521 |
| #4 | (("Stroke"[Mesh]) OR (((((((((((((((((((((((((apoplexy[Title/Abstract]) OR (Stroke, Ischemic[Title/Abstract])) OR (Ischaemic Stroke[Title/Abstract])) OR (Ischaemic Strokes[Title/Abstract])) OR (Stroke, Ischaemic[Title/Abstract])) OR (Cryptogenic Ischemic Stroke[Title/Abstract])) OR (Cryptogenic Ischemic Strokes[Title/Abstract])) OR (Ischemic Stroke, Cryptogenic[Title/Abstract])) OR (Stroke, Cryptogenic Ischemic[Title/Abstract])) OR (Cryptogenic Stroke[Title/Abstract])) OR (Cryptogenic Strokes[Title/Abstract])) OR (Stroke, Cryptogenic[Title/Abstract])) OR (Cryptogenic Embolism Stroke[Title/Abstract])) OR (Cryptogenic Embolism Strokes[Title/Abstract])) OR (Embolism Stroke, Cryptogenic[Title/Abstract])) OR (Stroke, Cryptogenic Embolism[Title/Abstract])) OR (Wake-up Stroke[Title/Abstract])) OR (Stroke, Wake-up[Title/Abstract])) OR (Wake up Stroke[Title/Abstract])) OR (Wake-up Strokes[Title/Abstract])) OR (Acute Ischemic Stroke[Title/Abstract])) OR (Acute Ischemic Strokes[Title/Abstract])) OR (Ischemic Stroke, Acute[Title/Abstract])) OR (Stroke, Acute Ischemic[Title/Abstract])) OR (cerebral injury[Title/Abstract]))) OR (Stroke[Title/Abstract]) | 394,963 |
| #5 | (("Deglutition Disorders"[Mesh]) OR (((((((((((deglutition disorder[Title/Abstract]) OR (Swallowing Disorders[Title/Abstract])) OR (swallowing disorder[Title/Abstract])) OR (Swallowing Difficulty[Title/Abstract])) OR (Difficulty Swallowing[Title/Abstract])) OR (Impaired Swallowing[Title/Abstract])) OR (Swallowing Dysfunction[Title/Abstract])) OR (Oropharyngeal Dysphagia[Title/Abstract])) OR (esophageal dysphagia[Title/Abstract])) OR (dysphagia[Title/Abstract])) OR (dysphagias[Title/Abstract]))) OR (Deglutition Disorders[Title/Abstract]) | 85,311 |
| #6 | (("Electroacupuncture"[Mesh]) OR ((((((Electro-acupuncture[Title/Abstract]) OR (Electric acupuncture[Title/Abstract])) OR (Electrical acupuncture[Title/Abstract])) OR (EA[Title/Abstract])) OR (Electric needle therapy[Title/Abstract])) OR (Electrostimulation acupuncture[Title/Abstract]))) OR (Electroacupuncture[Title/Abstract]) | 36,124 |
| #7 | ((randomized controlled trial[Publication Type]) OR (randomized[Title/Abstract])) OR (placebo[Title/Abstract]) | 113,581 |
| #8 | #4 AND #5 | 3475 |
| #9 | #8 AND #6 AND #7 | 20 |

**Table 2:** Embase Search

| **NO** | **Search Details** | **Results** |
| --- | --- | --- |
| #1 | stroke:ab,ti OR apoplexy:ab,ti OR 'stroke, ischemic':ab,ti OR 'ischaemic stroke':ab,ti OR 'ischaemic strokes':ab,ti OR 'stroke, ischaemic':ab,ti OR 'cryptogenic ischemic stroke':ab,ti OR 'cryptogenic ischemic strokes':ab,ti OR 'ischemic stroke, cryptogenic':ab,ti OR 'stroke, cryptogenic ischemic':ab,ti OR 'cryptogenic stroke':ab,ti OR 'cryptogenic strokes':ab,ti OR 'stroke, cryptogenic':ab,ti OR 'cryptogenic embolism stroke':ab,ti OR 'cryptogenic embolism strokes':ab,ti OR 'embolism stroke, cryptogenic':ab,ti OR 'stroke, cryptogenic embolism':ab,ti OR 'wake-up stroke':ab,ti OR 'stroke, wake-up':ab,ti OR 'wake up stroke':ab,ti OR 'wake-up strokes':ab,ti OR 'acute ischemic stroke':ab,ti OR 'acute ischemic strokes':ab,ti OR 'ischemic stroke, acute':ab,ti OR 'troke, acute ischemic':ab,ti OR 'cerebral injury':ab,ti | 546974 |
| #2 | 'deglutition disorders':ab,ti OR 'deglutition disorder':ab,ti OR 'swallowing disorders':ab,ti OR 'swallowing disorder':ab,ti OR 'swallowing difficulty':ab,ti OR 'difficulty swallowing':ab,ti OR 'impaired swallowing':ab,ti OR 'swallowing dysfunction':ab,ti OR 'oropharyngeal dysphagia':ab,ti OR 'esophageal dysphagia':ab,ti OR dysphagia:ab,ti OR dysphagias:ab,ti | 71160 |
| #3 | electroacupuncture:ab,ti OR 'electro acupuncture':ab,ti OR 'electric acupuncture':ab,ti OR 'electrical acupuncture':ab,ti OR ea:ab,ti OR 'electrostimulation acupuncture':ab,ti | 40233 |
| #4 | 'randomized controlled trial':it OR randomized:ab,ti OR placebo:ab,ti | 1255303 |
| #5 | #1 AND #2 | 5190 |
| #6 | #5 AND #3 AND #4 | 13 |

**Table 3:** Web of Science Search

| **NO** | **Search Details** | **Results** |
| --- | --- | --- |
| #1 | TS=(Stroke OR apoplexy OR Stroke, Ischemic OR Ischaemic Stroke OR Ischaemic Strokes Stroke, Ischaemic OR Cryptogenic Ischemic Stroke OR Cryptogenic Ischemic Strokes Ischemic Stroke, Cryptogenic OR Stroke, Cryptogenic Ischemic OR Cryptogenic Stroke Cryptogenic Strokes OR Stroke, Cryptogenic OR Cryptogenic Embolism Stroke OR Cryptogenic Embolism Strokes OR Embolism Stroke, Cryptogenic OR Stroke, Cryptogenic Embolism OR Wake-up Stroke OR Stroke, Wake-up OR Wake up Stroke OR Wake-up Strokes OR Acute Ischemic Stroke OR Acute Ischemic Strokes OR Ischemic Stroke, Acute OR Stroke, Acute Ischemic OR cerebral injury) | 799814 |
| #2 | TS=(deglutition disorders OR deglutition disorde OR Swallowing Disorders OR swallowing disorder OR Swallowing Difficulty OR Difficulty Swallowing OR Impaired Swallowing OR Swallowing Dysfunction OR Oropharyngeal Dysphagia OR esophageal dysphagia OR dysphagia OR dysphagias) | 80029 |
| #3 | TS=(Electroacupuncture OR Electro-acupuncture OR Electric acupuncture OR Electrical acupuncture OR EA OR Electric needle therapy OR Electrostimulation acupuncture) | 84071 |
| #4 | TS=(randomized controlled trial OR randomized OR placebo) | 1611368 |
| #5 | #1 AND #2 | 7105 |
| #6 | #5 AND #3 AND #4 | 37 |

**Table 4:** Cochrane Library Search

| **NO** | **Search Details** | **Results** |
| --- | --- | --- |
| #1 | MeSH descriptor: [Stroke] explode all trees | 17720 |
| #2 | MeSH descriptor: [Deglutition Disorders] explode all trees | 4102 |
| #3 | MeSH descriptor: [Electroacupuncture] explode all trees | 1170 |
| #4 | （Stroke OR apoplexy OR Stroke, Ischemic OR Ischaemic Stroke OR Ischaemic Strokes OR Stroke, Ischaemic OR Cryptogenic Ischemic Stroke OR Cryptogenic Ischemic Strokes OR Ischemic Stroke, Cryptogenic OR Stroke, Cryptogenic Ischemic OR Cryptogenic Stroke OR Cryptogenic Strokes OR Stroke, Cryptogenic OR Cryptogenic Embolism Stroke OR Cryptogenic Embolism Strokes OR Embolism Stroke, Cryptogenic OR Stroke, Cryptogenic Embolism OR Wake-up Stroke OR Stroke, Wake-up OR Wake up Stroke OR Wake-up Strokes OR Acute Ischemic Stroke OR Acute Ischemic Strokes OR Ischemic Stroke, Acute OR Stroke, Acute Ischemic OR cerebral injury ）:ab,ti,kw | 91264 |
| #5 | (deglutition disorders OR deglutition disorde OR Swallowing Disorders OR swallowing disorder OR Swallowing Difficulty OR Difficulty Swallowing OR Impaired Swallowing OR Swallowing Dysfunction OR Oropharyngeal Dysphagia OR esophageal dysphagia OR dysphagia OR dysphagias):ab,ti,kw | 7281 |
| #6 | (Electroacupuncture OR Electro-acupuncture OR Electric acupuncture OR Electrical acupuncture OR EA OR Electric needle therapy OR Electrostimulation acupuncture):ab,ti,kw | 7956 |
| #7 | (randomized controlled trial OR randomized OR placebo):ab,ti,kw | 1328348 |
| #8 | #1 OR #4 | 91652 |
| #9 | #2 OR #5 | 9726 |
| #10 | #3 OR #6 | 7956 |
| #11 | #8 AND #9 | 1510 |
| #12 | #11 AND #10 AND #7 | 31 |

**Table 5:** CNKI Search

| **NO** | **Search Details** | **Results** |
| --- | --- | --- |
| #1 | （主题：卒中 + 中风 + 脑梗死 + 脑栓塞 + 脑血栓 + 脑血管闭塞 + 脑血管意外） | 353427 |
| #2 | （主题：吞咽困难 + 吞咽障碍 + 吞咽功能障碍） | 24339 |
| #3 | （主题：电针 + 电针疗法 + 电针治疗 + 电针干预 + 电针刺激） | 43561 |
| #4 | （摘要：随机对照试验 + 随机对照研究 + 随机对照 + 随机 + RCT(精确)） | 2597518 |
| #5 | #1 and #2 and #3 and #4 | 185 |

**Table 6:** Wanfang Search

| **NO** | **Search Details** | **Results** |
| --- | --- | --- |
| #1 | 主题:(卒中 or 中风 or 脑梗死 or 脑栓塞 or 脑血栓 or 脑血管闭塞 or 脑血管意外) | 467715 |
| #2 | 主题:(吞咽困难 or 吞咽障碍 or 吞咽功能障碍) | 36457 |
| #3 | 主题:(电针 or 电针疗法 or 电针治疗 or 电针干预 or 电针刺激) | 39192 |
| #4 | 主题:(随机对照试验 or 随机对照研究 or 随机对照 or 随机 or RCT) | 3519508 |
| #5 | #1 and #2 and #3 and #4 | 211 |

**Table 7:**VIP Search

| **NO** | **Search Details** | **Results** |
| --- | --- | --- |
| #1 | [((((((题名或关键词=卒中 OR 题名或关键词=中风) OR 题名或关键词=脑梗死) OR 题名或关键词=脑栓塞) OR 题名或关键词=脑血栓) OR 题名或关键词=脑血管闭塞) OR 题名或关键词=脑血管意外)](https://qikan.cqvip.com/Qikan/search/index?LngMySearHistoryIdGuid=50ed86a2-d672-4460-9f21-0d591c106cc0&from=Qikan_Article_History) | 283276 |
| #2 | [((题名或关键词=吞咽困难 OR 题名或关键词=吞咽障碍) OR 题名或关键词=吞咽功能障碍)](https://qikan.cqvip.com/Qikan/search/index?LngMySearHistoryIdGuid=9caa78e3-2f1a-483d-898b-9f7e2618945f&from=Qikan_Article_History) | 15932 |
| #3 | [((((题名或关键词=电针 OR 题名或关键词=电针疗法) OR 题名或关键词=电针治疗) OR 题名或关键词=电针干预) OR 题名或关键词=电针刺激)](https://qikan.cqvip.com/Qikan/search/index?LngMySearHistoryIdGuid=cebd304e-f720-46ee-b783-cab1554ccb53&from=Qikan_Article_History) | 23552 |
| #4 | [((((摘要=随机对照试验 OR 摘要=随机对照研究) OR 摘要=随机对照) OR 摘要=随机) OR 摘要=RCT)](https://qikan.cqvip.com/Qikan/search/index?LngMySearHistoryIdGuid=7243d770-2320-418a-882b-746de06848d1&from=Qikan_Article_History) | 2517027 |
| #5 | #1 and #2 and #3 and #4 | 114 |

**Table 8:** CBM Search

| NO | Search Details | Results |
| --- | --- | --- |
| #1 | ["卒中"[常用字段:智能] OR "中风"[常用字段:智能] OR "脑梗死"[常用字段:智能] OR "脑栓塞"[常用字段:智能] OR "脑血栓"[常用字段:智能] OR "脑血管闭塞"[常用字段:智能] OR "脑血管意外"[常用字段:智能]](javascript:toDoRelimitSearch();) | 568565 |
| #2 | ["吞咽困难"[常用字段:智能] OR "吞咽障碍"[常用字段:智能] OR "吞咽功能障碍"[常用字段:智能]](javascript:toDoRelimitSearch();) | 35918 |
| #3 | ["电针"[常用字段:智能] OR "电针疗法"[常用字段:智能] OR "电针治疗"[常用字段:智能] OR "电针干预"[常用字段:智能] OR "电针刺激"[常用字段:智能]](javascript:toDoRelimitSearch();) | 29456 |
| #4 | ["随机对照试验"[摘要:智能] OR "随机对照研究"[摘要:智能] OR "随机对照"[摘要:智能] OR "随机"[摘要:智能] OR "RCT"[摘要:智能]](javascript:toDoRelimitSearch();) | 2025180 |
| #5 | #1 and #2 and #3 and #4 | 174 |
