# Supplementary material for: The effect and optimal parameters of electroacupuncture on post-stroke dysphagia: a meta-analysis of randomized controlled trials
Source: Front Neurol. 2026 Jan 12;16:1673716. doi: 10.3389/fneur.2025.1673716 (PMC12832666; doi:10.3389/fneur.2025.1673716)
Supplement: Supplementary file 3 [file Table_3.docx]

**Identification of studies via databases and registers**

Literature obtained by searching databases (N=785)

Duplicate records removed (n =393 )

**Identification**

Exclude Meta-analysis, systematic review, animal experiments, and master and doctoral conference papers(N=40)

Literatures obtained after primary screening(N=392)

Literatures obtained after primary screening(N=352)

Not relevant(N=300)

**Screening**

Article included after full text review(N=52)

Reports excluded:

1.Duplicate data(N=7)

2.Incomplete data(N=15)

The final included literature(N=30)

**Included**

*Consider, if feasible to do so, reporting the number of records identified from each database or register searched (rather than the total number across all databases/registers).

**If automation tools were used, indicate how many records were excluded by a human and how many were excluded by automation tools.

*From:*  Page MJ, McKenzie JE, Bossuyt PM, Boutron I, Hoffmann TC, Mulrow CD, et al. The PRISMA 2020 statement: an updated guideline for reporting systematic reviews. BMJ 2021;372:n71. doi: 10.1136/bmj.n71

For more information, visit: <http://www.prisma-statement.org/>
