# Supplementary material for: The effect and optimal parameters of electroacupuncture on post-stroke dysphagia: a meta-analysis of randomized controlled trials
Source: Front Neurol. 2026 Jan 12;16:1673716. doi: 10.3389/fneur.2025.1673716 (PMC12832666; doi:10.3389/fneur.2025.1673716)
Supplement: Supplementary file 4 [file Table_4.docx]

***Supplementary Material***

**Supplementary File 4** grade pro assessment results

| **EA FOR PSD** | | | | | | | |
| --- | --- | --- | --- | --- | --- | --- | --- |
| **Patient or population:** patients with PSD **Settings:**  **Intervention:** EA FOR PSD | | | | | | | |
| **Outcomes** | **Illustrative comparative risks* (95% CI)** | | | **Relative effect (95% CI)** | **No of Participants (studies)** | **Quality of the evidence (GRADE)** | **Comments** |
|  | Assumed risk | Corresponding risk | |  |  |  |  |
|  | **Control** | **EA FOR PSD** | |  |  |  |  |
| **VFSS change** |  | The mean vfss change in the intervention groups was **1.67 higher** (1.26 to 2.09 higher) | |  | 496 (8 studies) | ⊕⊕⊕⊕ **high** |  |
| **WST change** |  | The mean wst change in the intervention groups was **0.75 lower** (0.93 to 0.57 lower) | |  | 636 (9 studies) | ⊕⊕⊕⊕ **high** |  |
| **Adverse events** | **Study population** | | | **RR 0.41**  (0.25 to 0.68) | 406 (5 studies) | ⊕⊕⊕⊕ **high** |  |
|  | **208 per 1000** | | **85 per 1000** (52 to 141) |  |  |  |  |
|  | **Moderate** | |  |  |  |  |  |
|  | **222 per 1000** | | **91 per 1000** (56 to 151) |  |  |  |  |
| **Effective rate** | **Study population** | |  | **RR 1.29**  (2.05 to 3.16) | 1933 (24 studies) | ⊕⊕⊕⊝ **moderate**^1^ |  |
|  | **716 per 1000** | | **85 per 1000** (52 to 141) |  |  |  |  |
|  | **Moderate** | |  |  |  |  |  |
|  | **712 per 1000** | | **918 per 1000** (876 to 954) |  |  |  |  |
| **Time** | **Study population** | | | **RR 1.27**  (1.21 to 1.33) | 1851 (23 studies) | ⊕⊕⊕⊕ **high** |  |
|  | **711 per 1000** | | **903 per 1000** (860 to 945) |  |  |  |  |
|  | **Moderate** | | |  |  |  |  |
|  | **706 per 1000** | | **897 per 1000** (854 to 939) |  |  |  |  |
| **Time:＜30min** | **Study population** | | | **RR 1.38**  (1.18 to 1.63) | 336 (4 studies) | ⊕⊕⊕⊝ **moderate**^2^ |  |
|  | **661 per 1000** | | **912 per 1000** (780 to 1000) |  |  |  |  |
|  | **Moderate** | | |  |  |  |  |
|  | **715 per 1000** | | **987 per 1000** (844 to 954) |  |  |  |  |
| **Time:≥30min** | **Study population** | | | **RR 1.24**  (1.19 to 1.3) | 1515 (19 studies) | ⊕⊕⊕⊕ **high** |  |
|  | **722 per 1000** | | **895 per 1000** (859 to 938) |  |  |  |  |
|  | **Moderate** | | |  |  |  |  |
|  | **700 per 1000** | | **868 per 1000** (833 to 910) |  |  |  |  |
| **Waveform** | **Study population** | | | **RR 1.3**  (1.24 to 1.36) | 1699 (21 studies) | ⊕⊕⊕⊕ **high** |  |
|  | **713 per 1000** | | **927 per 1000** (884 to 970) |  |  |  |  |
|  | **Moderate** | | |  |  |  |  |
|  | **711 per 1000** | | **924 per 1000** (882 to 967) |  |  |  |  |
| **Waveform:Ds-W** | **Study population** | | | **RR 1.58**  (1.39 to 1.81) | 326 (5 studies) | ⊕⊕⊕⊕ **high** |  |
|  | **593 per 1000** | | **936 per 1000** (824 to 1000) |  |  |  |  |
|  | **Moderate** | | |  |  |  |  |
|  | **667 per 1000** | | **1000 per 1000** (927 to 1000) |  |  |  |  |
| **Waveform:C-W** | **Study population** | | | **RR 1.23**  (1.16 to 1.31) | 828 (10 studies) | ⊕⊕⊕⊕ **high** |  |
|  | **752 per 1000** | | **925 per 1000** (873 to 986) |  |  |  |  |
|  | **Moderate** | | |  |  |  |  |
|  | **750 per 1000** | | **922 per 1000** (870 to 982) |  |  |  |  |
| **Waveform:I-W** | **Study population** | | | **RR 1.26**  (1.16 to 1.36) | 545 (6 studies) | ⊕⊕⊕⊕ **high** |  |
|  | **725 per 1000** | | **914 per 1000** (841 to 986) |  |  |  |  |
|  | **Moderate** | | |  |  |  |  |
|  | **712 per 1000** | | **897 per 1000** (826 to 968) |  |  |  |  |
| **Frequency** | **Study population** | | | **RR 1.22**  (1.16 to 1.29) | 1095 (12 studies) | ⊕⊕⊕⊕ **high** |  |
|  | **751 per 1000** | | **916 per 1000** (871 to 969) |  |  |  |  |
|  | **Moderate** | | |  |  |  |  |
|  | **740 per 1000** | | **903 per 1000** (858 to 955) |  |  |  |  |
| **Frequency:≤5HZ** | **Study population** | | | **RR 1.2**  (1.14 to 1.27) | 943 (10 studies) | ⊕⊕⊕⊕ **high** |  |
|  | **762 per 1000** | | **915 per 1000** (869 to 968) |  |  |  |  |
|  | **Moderate** | | |  |  |  |  |
|  | **763 per 1000** | | **916 per 1000** (870 to 969) |  |  |  |  |
| **Frequency:≥30HZ** | **Study population** | | | **RR 1.36**  (1.14 to 1.6) | 152 (2 studies) | ⊕⊕⊕⊝ **moderate**^2^ |  |
|  | **680 per 1000** | | **925 per 1000** (775 to 1000) |  |  |  |  |
|  | **Moderate** | | |  |  |  |  |
|  | **678 per 1000** | | **922per 1000** (773 to 1000) |  |  |  |  |
| **Electroacupuncture parameter combination** | **Study population** | | | **RR 1.3**  (1.23 to 1.37) | 943 (10 studies) | ⊕⊕⊕⊕ **high** |  |
|  | **715 per 1000** | | **929 per 1000** (879 to 979) |  |  |  |  |
|  | **Moderate** | | |  |  |  |  |
|  | **700 per 1000** | | **905 per 1000** (856 to 954) |  |  |  |  |
| **Combination:≥30min+Ds-W** | **Study population** | | | **RR 1.55**  (1.32 to 1.82) | 226 (4 studies) | ⊕⊕⊕⊝ **moderate**^2^ |  |
|  | **598 per 1000** | | **927 per 1000** (790 to 907) |  |  |  |  |
|  | **Moderate** | | |  |  |  |  |
|  | **606 per 1000** | | **939 per 1000** (800 to 1000) |  |  |  |  |
| **Combination:≥30min+I-W** | **Study population** | | | **RR 1.28**  (1.17 to 1.4) | 447 (5 studies) | ⊕⊕⊕⊕ **high** |  |
|  | **714 per 1000** | | **914 per 1000** (836 to 1000) |  |  |  |  |
|  | **Moderate** | | |  |  |  |  |
|  | **700 per 1000** | | **896 per 1000** (819 to 980) |  |  |  |  |
| **Combination:≥30min+C-W** | **Study population** | | | **RR 1.23**  (1.15 to 1.32) | 608  (7 studies) | ⊕⊕⊕⊕ **high** |  |
|  | **758 per 1000** | | **933 per 1000** (872 to 1000) |  |  |  |  |
|  | **Moderate** | | |  |  |  |  |
|  | **766 per 1000** | | **942 per 1000** (881 to 1000) |  |  |  |  |
| *The basis for the **assumed risk** (e.g. the median control group risk across studies) is provided in footnotes. The **corresponding risk** (and its 95% confidence interval) is based on the assumed risk in the comparison group and the **relative effect** of the intervention (and its 95% CI).  **CI:** Confidence interval; **RR:** Risk ratio; | | | | | | | |
| GRADE Working Group grades of evidence **High quality:** Further research is very unlikely to change our confidence in the estimate of effect.  **Moderate quality:** Further research is likely to have an important impact on our confidence in the estimate of effect and may change the estimate. **Low quality:** Further research is very likely to have an important impact on our confidence in the estimate of effect and is likely to change the estimate. **Very low quality:** We are very uncertain about the estimate. | | | | | | | |
| ^1^ There is suspicion of publication bias. ^2^ The sample size is insufficient. | | | | | | | |
