# Supplementary figures and images for: The effect and optimal parameters of electroacupuncture on post-stroke dysphagia: a meta-analysis of randomized controlled trials
Source: Front Neurol. 2026 Jan 12;16:1673716. doi: 10.3389/fneur.2025.1673716 (PMC12832666; doi:10.3389/fneur.2025.1673716)

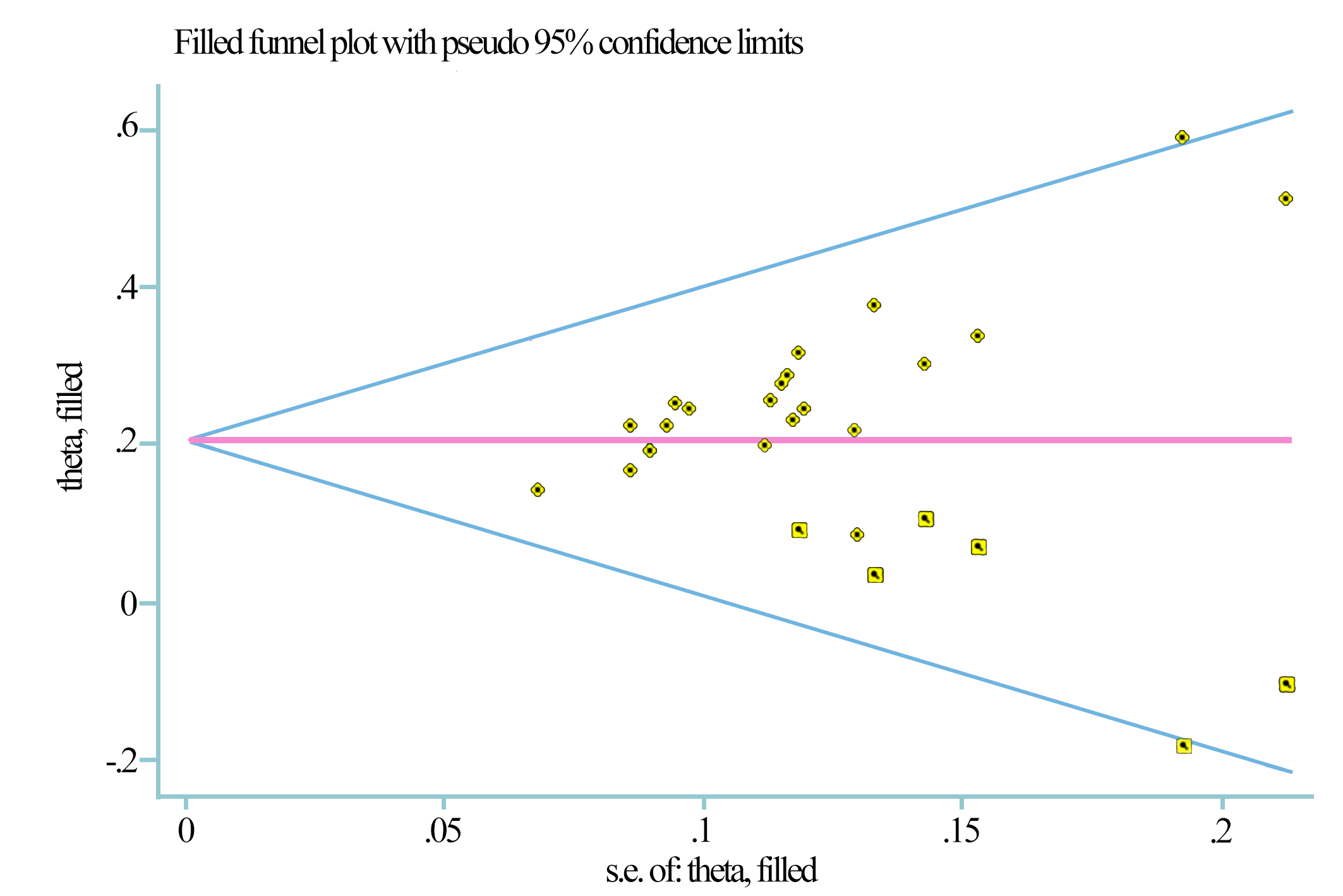

Supplement: Supplementary file 5 [file Image_1.tif]
